# Ochre-based compound adhesives at the Mousterian type-site document complex cognition and high investment

**DOI:** 10.1126/sciadv.adl0822

**Published:** 2024-02-21

**Authors:** Patrick Schmidt, Radu Iovita, Armelle Charrié-Duhaut, Gunther Möller, Abay Namen, Ewa Dutkiewicz

**Affiliations:** ^1^ Early Prehistory and Quaternary Ecology, Department of Geosciences, Eberhard Karls University of Tübingen, Tübingen, Germany.; ^2^ Applied Mineralogy, Department of Geosciences, Eberhard Karls University of Tübingen, Tübingen, Germany.; ^3^ Center for the Study of Human Origins, Department of Anthropology, New York University, New York, NY 10003, USA.; ^4^ Laboratoire de spectrométrie de masse des interactions et des systèmes (LSMIS), Strasbourg University, CNRS, CMC UMR, Strasbourg 7140, France.; ^5^ Department of Sociology and Anthropology, School of Sciences and Humanities, Nazarbayev University, Astana, Kazakhstan.; ^6^ Staatliche Museen zu Berlin, Museum für Vor- und Frühgeschichte, Berlin, Germany.

## Abstract

Ancient adhesives used in multicomponent tools may be among our best material evidences of cultural evolution and cognitive processes in early humans. African *Homo sapiens* is known to have made compound adhesives from naturally sticky substances and ochre, a technical behavior proposed to mark the advent of elaborate cognitive processes in our species. Foragers of the European Middle Paleolithic also used glues, but evidence of ochre-based compound adhesives is unknown. Here, we present evidence of this kind. Bitumen was mixed with high loads of goethite ochre to make compound adhesives at the type-site of the Mousterian, Le Moustier (France). Ochre loads were so high that they lowered the adhesive’s performance in classical hafting situations where stone implements are glued to handles. However, when used as handheld grips on cutting or scraping tools, a behavior known from Neanderthals, high-ochre adhesives present a real benefit, improving their solidity and rigidity. Our findings help understand the implications of Pleistocene adhesive making.

## Teaser

The oldest ochre-based compound adhesives of Europe were found at Le Moustier, highlighting similarities with the African record.

## INTRODUCTION

In archaeology, evolutionary concepts like modern behaviors (*1*) or complex cognition (*2*) have been argued for based on tool (*3*) and raw material behaviors (*4*), the transformation of materials (*5*, *6*), and composite technology or hafting (*7*, *8*). Adhesives play an important role in arguments about the implications of hafting because, in some instances, Stone Age foragers used elaborate production techniques to make them (*9*–*11*). Adhesive making may therefore contain information about innovative behavior, social learning, and cumulative culture (*2*, *5*, *12*). In the African Middle Stone Age (MSA) [~300 to 30 thousand years (ka) ago], this debate was fueled by the finding that *Homo sapiens* combined naturally sticky materials with other ingredients, such as ochre (*13*, *14*) or bone fragments and quartz (*15*), to produce adhesives with properties not otherwise available in nature (*16*, *17*). Some researchers (*18*) argue that mixing substances following specific recipes requires analogical reasoning and forward planning. If so, compound adhesives might be one of our best indicators of elaborate cognitive processes in human evolution. In Eurasia, Neanderthals also used naturally available adhesives, such as bitumen (*19*) or tree resins [in one case possibly mixed with beeswax (*20*)]. In some instances, they made tar by distilling birch bark (*5*, *21*, *22*). European *H. sapiens* also used adhesives from the late Aurignacian and Gravettian (<33 ka) on (*23*–*25*), but their botanical origin and production techniques are not well understood [an exception is the use of bitumen in Eastern Europe (*26*)]. The implications of Neanderthal birch tar are still debated [compare (*27*, *28*)], but what is certain is that Neanderthals made the effort to produce it, although other adhesive substances could simply have been collected in nature [see the discussion in (*9*, *29*, *30*)]. Thus, similarly to the modern human record from Africa, the known European adhesive technology documents innovative behavior and even cumulative cultural transmission of techniques (*31*, *32*). In addition, from the Gravettian on and in later periods, there are indications that European *H. sapiens* made compound adhesives using ochre (*24*, *25*), a behavior similar to that of early *H. sapiens* in Africa. However, neither Neanderthals nor early *H. sapiens* present in Europe before 40 ka (*33*) have been shown to make ochre-based compound adhesives like those known from Africa (*34*). Therefore, the question whether early European adhesives from the Middle Paleolithic have the same cognitive implications as those made in the African MSA remains unanswered.

Here, we report on the discovery of such ochre-based multicomponent adhesives. For this, we analyze so far unstudied artifacts from the type-site of the Mousterian technocomplex (Le Moustier, France) that are curated at the Museum für Vor- und Frühgeschichte in Berlin (Germany). The Berlin Le Moustier collection was excavated at the upper terrace of Le Moustier shelter by Swiss archaeologist Otto Hauser in 1907. Because these artifacts have never been studied before and even remained individually packed and untouched since the 1960s, they allow unique preservation conditions for organic residues.

## RESULTS

**Fig. 1.**
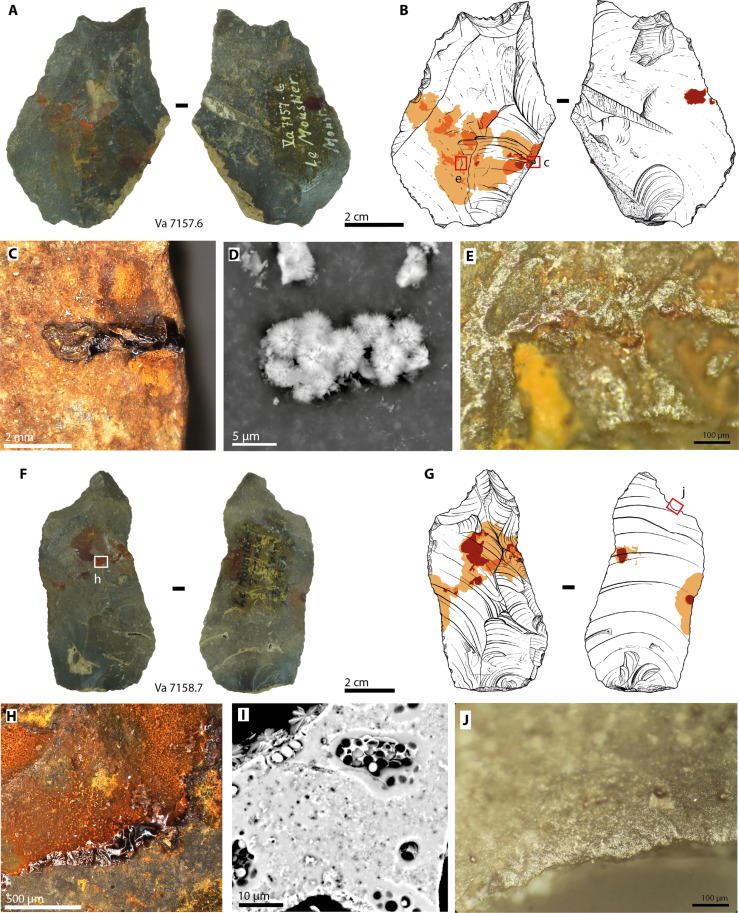
Photographs, drawings, and details of Le Moustier artifacts nos. Va 7157.6 and Va 7158.7. (**A**) Photographs of dorsal and ventral surfaces of artifact Va 7157.6. (**B**) Drawing showing the distribution of residue remains on both sides of the artifact. The red box marks the frame of the detail shown in (C). (**C**) Detail of artifact no. Va 7157.6 showing an adhesive protuberance. (**D**) Scanning electron micrograph of spherulites constituting the powder removed from artifact no. Va 7157.6. (**E**) Micrograph showing polish caused by abrasion in the presumed prehensile portion of the tool. (**F**) Photographs of dorsal and ventral surfaces of artifact no. Va 7158.7. (**G**) Drawing showing the repartition of residue remains on both sides of the artifact. (**H**) Detail of artifact no. Va 7158.7 showing adhesive residues. (**I**) Scanning electron micrograph of the residue removed from artifact no. Va 7158.7. Note the spherulites on top of the micrograph and air bubbles trapped in the solid adhesive mass. (**J**) Micrograph showing invasive use wear polish in the presumed active edge of the tool. Drawings by D. Greinert.

We identified five lithic artifacts with traces of red and yellow colorants on their ventral and dorsal sides (Fig. 1). Typologically (*35*), one is an atypical Levallois flake (no. Va 7136), one an end-notched flake (no. Va 7157.6), one a retouched flake (no. Va 7167.9), one a retouched Levallois blade (no. Va 7158.7), and one a side scraper (no. Va 7157.24). In all cases, red and yellow stains are restricted to one portion of the tools, forming a wall effect that effectively separates the portions of the tools with stains from the clean opposite portions (Fig. 1, but also see figs. S1 to S5). Four of the flakes also show traces of a black residue associated with red colorants (table S1; Fig. 1, C and H; and fig. S6). In one case, black residue appears as a visually shiny, ~2.5-mm-long, and ~1-mm-high protuberance (Fig. 1C). The distribution of colorant stains and their association with black residue are consistent with one portion of the pieces being covered by an adhesive containing red colorant. The opposite portion would then have acted as the active part of a composite tool. To test this hypothesis, we investigated the pieces for traces of wear. We found micro-fractures and localized polishes on the presumed active edges of four tools (Fig. 1J and figs. S1 to S5), documenting that they were used. Moreover, we found bright polish and striations under colorant stains, away from the edges, on dorsal and ventral faces of all five tools (Fig. 1E and figs. S1 and S3 to S5). Parts of the raised topography (the ridges of previous removal scars) are abraded in these zones. Such polishes, which are typical of hafting wear (*36*–*38*), are found on relatively large areas across the entire surface of the presumed hafted portion of the tools but not outside of it, and not only at the limit of the residue. We interpret these observations as evidence that the tools were held in an adhesive that still allowed movement of the stone tools or that underwent plastic deformation, with the colorant acting as an abrasive agent. The restriction of polished zones and striations to the presumed hafted parts rules out postdepositional processes as their cause (*39*). These findings support the hypothesis that the Le Moustier artifacts were part of composite tools. They were assembled by gluing them to shafts or handles using an adhesive, or alternatively, as Neanderthal archaeological examples show (*22*, *27*), the adhesive was molded onto the tools and functioned as the handle itself.

### Chemical and structural analysis

**Fig. 2.**
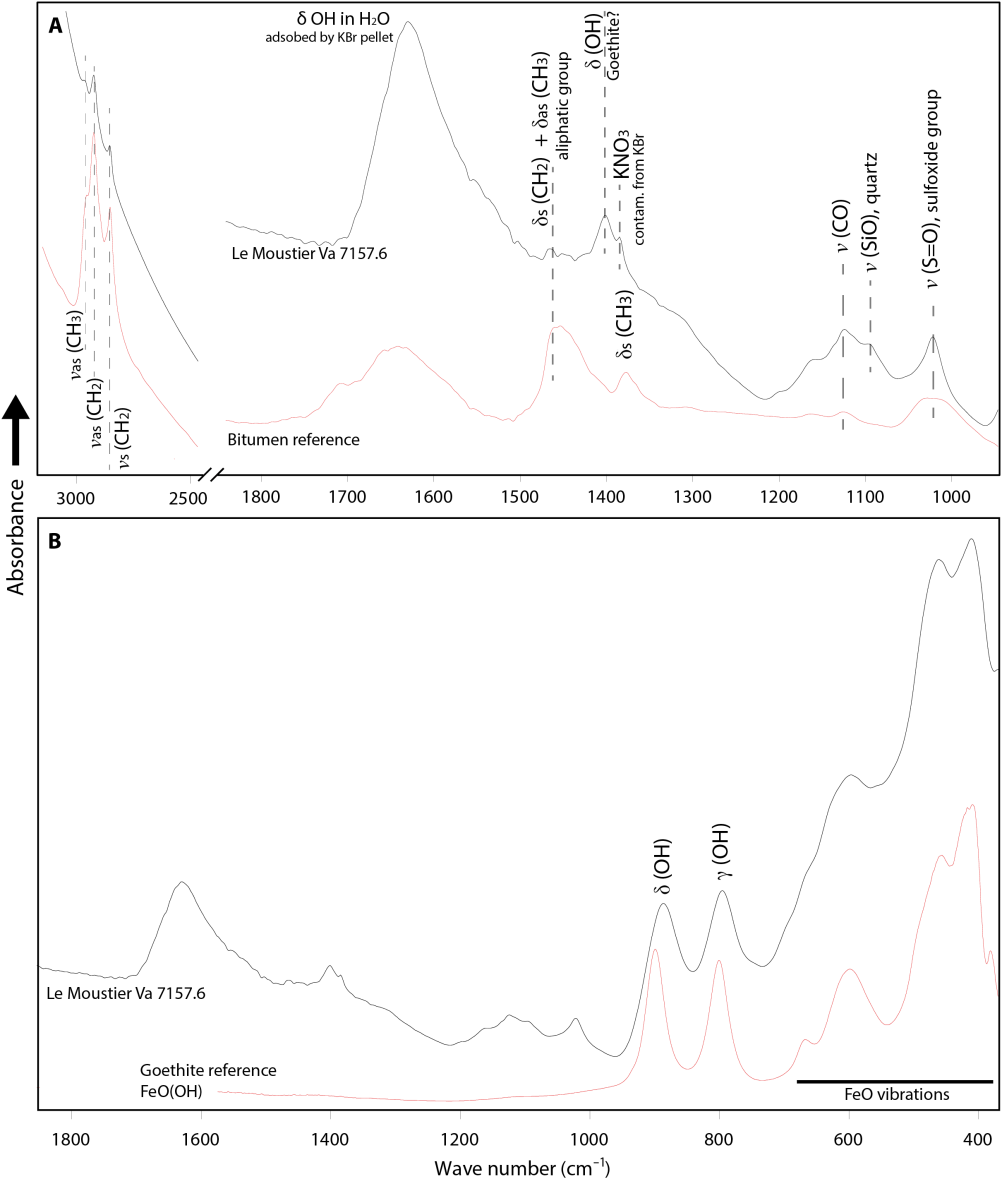
Transmission IR spectra of the Le Moustier adhesive (removed from artifact no. Va 7157.6) compared to reference spectra of known substances. The 0.3 g–weighing KBr pellets contained 1 mg of sample. (**A**) Comparison with a bitumen reference spectrum. (**B**) Comparison with a Goethite [α-FeO(OH)] reference spectrum. Note that the wave number region below ~1000 cm^−1^ of the Le Moustier spectrum can be explained by goethite being mixed with the adhesive. The wave number regions 3000 to 2500 cm^−1^ and 1800 to 1000 cm^−1^ are best explained by the insoluble fraction of bitumen (asphaltene). Note the absence of a C═O band between 1750 and 1700 cm^−1^ in the Le Moustier spectrum, which would be characteristic for plant-derived adhesives.

We first conducted energy-dispersive x-ray spectroscopy (EDX; using a scanning electron microscope) on powders scraped from the surface of two of the pieces (acc. nos. Va 7157.6 and Va 7158.7; Fig. 1, D and F, and figs. S9 and S10). The powders consist of ~5- to 10-μm-large spherulites that yield an EDX signal of iron and oxygen. Parts of the powders contain agglomerates held together by a substance between the spherulites that show no discernible structure but air bubbles (Fig. 1I). The EDX spectrum of the agglomerated parts shows sulfur and carbon in addition to iron and oxygen. To investigate what the spherulites and the adhesive itself are made of, we removed 1 mg of a protuberance where the adhesive is still intact (on acc. no. Va 7157.6, Fig. 1C) and analyzed it by transmission infrared (IR) spectroscopy. The spectral signature of the adhesive is consistent with an organic phase mixed with an inorganic filler (Fig. 2). The inorganic phase causes the strongest absorption bands of the IR spectrum. Two relatively sharp bands of the δ(OH) and γ(OH) vibrations of α-FeO(OH) (goethite) are observed at 890 and 800 cm^−1^ (*40*). The low-frequency envelope <700 cm^−1^ is consistent with Fe-O vibrations in α-FeO(OH) (*41*). Thus, the inorganic filler of the Le Moustier artifact is unambiguously goethite ochre. The organic fraction of the adhesive causes weak but sharp bands between 3000 and 2800 cm^−1^ that indicate symmetrical and asymmetrical ν(CH_2_) and ν(CH_3_) vibrations. The region between 1500 and 1000 cm^−1^ gives insight into the nature of this organic phase. The compound adhesive spectrum presents a weak band at 1458 cm^−1^ assigned to symmetrical δ(CH_2_) and asymmetrical δ(CH_3_) vibrations of the aliphatic group. This broad band is known from the asphaltene fraction of crude oil or bitumen (*42*). There are also a ν(CO) band at 1125 cm^−1^ and a ν(S=O) band of the sulfoxide group at 1021 cm^−1^. The assignment of the 1021-cm^−1^ band to sulfoxide is strengthened by the finding of sulfur using EDX. Both bands (1125 and 1021 cm^−1^) are also commonly present in the IR spectrum of asphaltene (*42*, *43*). Sulfur typically accounts for less than 0.1% of natural tree resins and ambers (*44*), supporting the attribution of the Le Moustier spectrum to asphaltene/bitumen. The interpretation of the region between 1430 and 1330 cm^−1^, where a symmetrical δ(CH_3_) vibration would be expected in asphaltene, is not straightforward. This is so because, in some goethite specimens, a hydroxyl bending vibration causes a weak band at 1401 cm^−1^ (*45*) overlying this asphaltene δ(CH_3_) vibration. The Le Moustier spectrum also presents a weak but sharp KNO_3_ band, either resulting from impurities of the KBr used for pelleting (*46*) or created by grinding the sample together with KBr powder during sample preparation. Thus, the organic fraction of the sample cannot be observed in this wave number region (the shape of the broad band observed near 1403 cm^−1^ is most likely the result of the convolution of the goethite hydroxyl band and that of the potassium nitrate contamination). Nonetheless, the organic region of the Le Moustier IR spectrum can be best explained by the insoluble fraction of bitumen (asphaltene). This interpretation is strengthened by the absence of a C═O band in the spectrum. Strong C═O bands between 1750 and 1700 cm^−1^ are characteristic of different esters, acids, and aldehydes. They would be prominent in the spectra of plant-derived adhesives like resins and their tars (*47*, *48*), including birch tar (*21*, *49*). The absence of such C═O bands in our adhesive spectrum and the finding of sulfoxide, which is absent in plant-derived resins and tars, make a botanical origin highly unlikely (fig. S7). To corroborate our finding that the Le Moustier residue is made from bitumen, we attempted to conduct gas chromatography–mass spectrometry (GC-MS) on the organic extractable fraction of the 1-mg sample used for IR spectroscopy. The attempt failed, not yielding a signal above the method’s detection limit (also see fig. S8). This is not in contradiction with our results obtained by IR. Bitumen is mainly constituted of asphaltenes and contains a soluble fraction of less than 3%, which would potentially be visible in GC-MS (*50*, *51*). Such low soluble content calls for larger GC-MS samples, which are not available on the Le Moustier artifacts. What is certain is that, if the residue consisted of birch tar or tree resin, both substantially more soluble, we would have found a specific di- or triterpenoid signature (*52*, *53*). We did not observe plant-derived terpenoids.

**Fig. 3.**
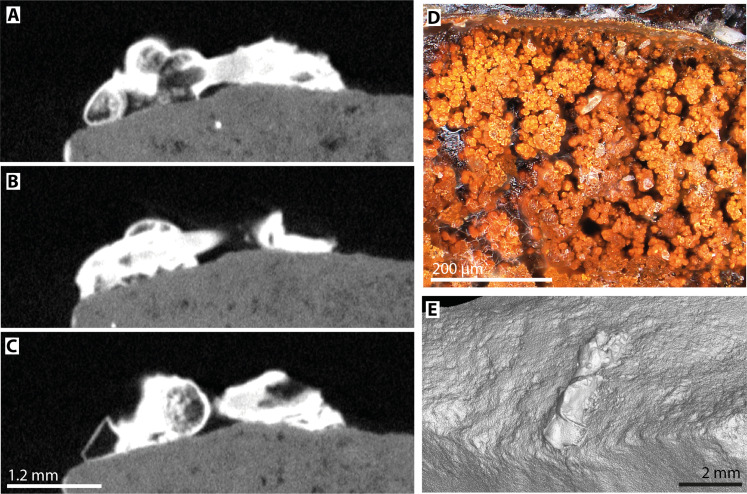
Inner structure of the adhesive protuberance on artifact no. Va 7157.6. (**A** to **C**) MicroCT scans through the long axis of the protuberance. Mark the air bubbles trapped in the adhesive. Around air bubbles, the adhesive appears homogeneous, indicating that goethite is evenly distributed. (**D**) Stereo-micrograph showing goethite spherulites in the compound adhesive. (**E**) Iso-surface recorded by the microCT scanner, showing the outer shape of the adhesive protuberance.

To gain information on the association of bitumen and goethite, we analyzed the adhesive protuberance on piece no. Va 7157.6 visually with a stereomicroscope. It is mostly opaque and black, but one portion, where it is broken, gives insight into its interior. Evenly distributed goethite spherulites can be seen there (Fig. 3D). To observe the inner structure of the Le Moustier residue, we recorded a micro–computed tomography (microCT) scan of the protuberance. On CT images, it shows no discernible goethite inclusions, as expected at the 11.5-μm resolution of our scan. Trapped air bubbles can be seen in the adhesive mass (Fig. 3, A to C). Around the bubbles, the adhesive’s structure appears mostly homogeneous with a few zones, where goethite distribution seems to be slightly lower. This is consistent with goethite being homogeneously mixed with bitumen. The adhesive mass has an overall brightness (organic part and ochre filler together) of 1539 Hounsfield units (HU). Assuming a roughly linear relationship between gray values in HU and density in our CT scans (*54*, *55*), the overall density of the adhesive can be calculated from its brightness value compared to the brightness value of the adjacent flint (1391 HU, for a known flint density of 2.58 g/cm^3^) as 2.85 g/cm^3^. Assuming a density of 1.01 g/cm^3^ of bitumen (*56*) and a density of 4.3 g/cm^3^ of α-FeO(OH) (*57*), the adhesive’s overall density of 2.85 g/cm^3^ implies that the compound adhesive was mixed from 55 and 45 wt % bitumen.

### Experimental testing of the ochre-bitumen mixture

**Fig. 4.**
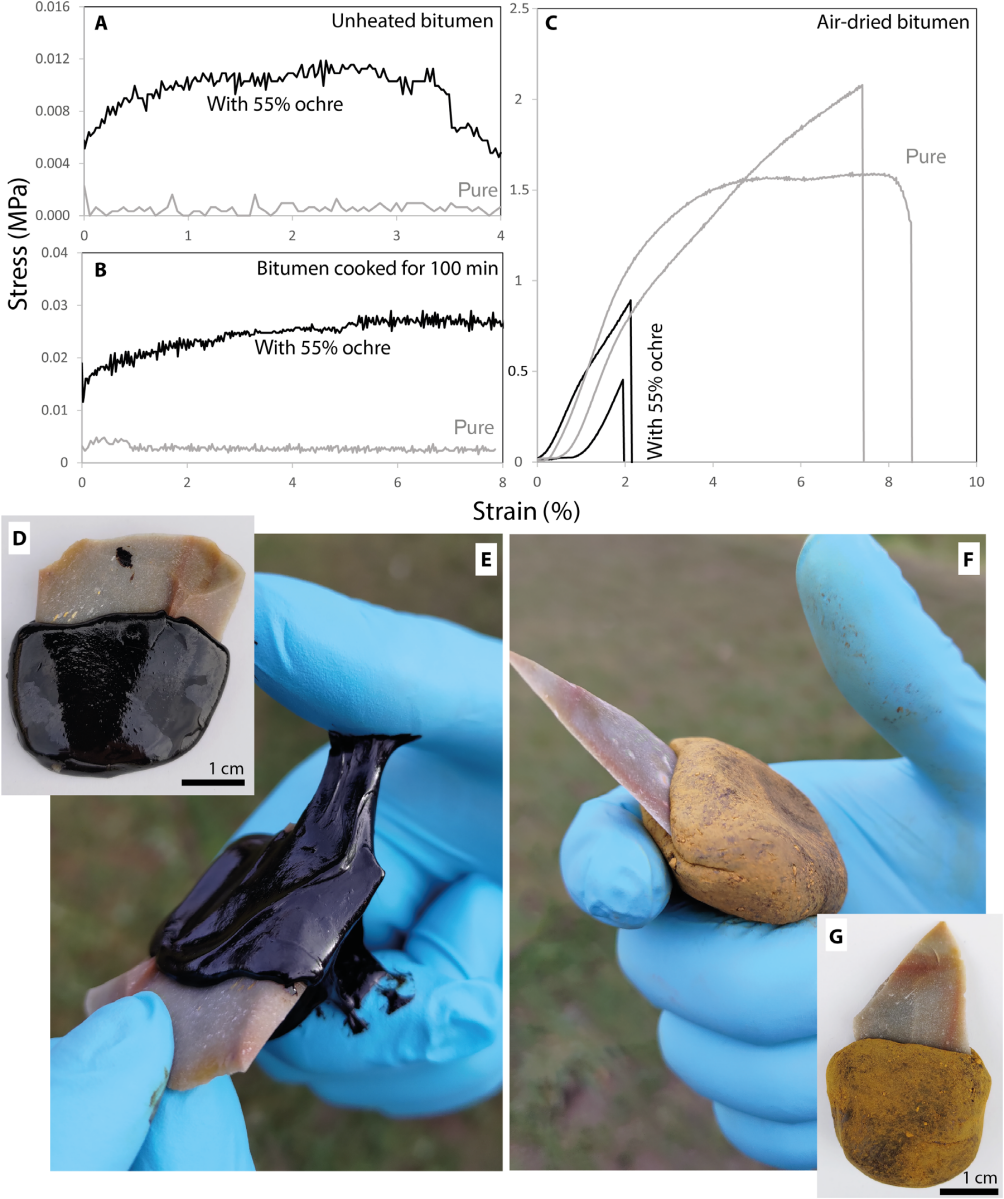
Experiments investigating the benefit of goethite ochre in bitumen-based adhesives. (**A** and **B**) Stress/strain diagrams of uncooked and cooked (100 min) fresh bitumen samples with and without ochre. (**C**) Stress/strain diagrams of air-dried bitumen with and without ochre. (**D** and **E**) Composite tool consisting of a stone tool and a grip made from fresh bitumen. (**F** and **G**) Composite tool consisting of a stone tool and a grip made from fresh bitumen and 55 wt % goethite ochre.

The proportion of filler to plastic component of the Le Moustier residue appears to be high in view of previous experiments conducted with inert filling loads in adhesive mixtures. Several experimental studies conducted on adhesives of different compositions show that ochre loading agents are beneficial if they account for 28% (*16*), 7.5 to 33% (*58*), or 10 to 30% (*59*, *60*) of the compound adhesives. A few experimental studies report data on two-component adhesives containing larger parts of inorganic filler than plastic material (*2*, *17*), but these studies investigated ochre mixed with *Vachellia* gum. To understand the usefulness of 55% goethite ochre in bitumen, we conducted lap-shear tests to measure adhesive strength (*61*), using naturally available bitumen (from the Massif Central region, France) and pure goethite ochre (from the Lessini Mountains, Italy). Natural bitumen can be collected in bituminous lakes as viscous liquid or as hardened air-dried mass on rock walls from where it seeps out (fig. S11). The viscosity of fresh bitumen is too low for laboratory strength testing. We therefore conducted a cooking experiment attempting to increase its adhesive strength (*62*). A subsample was removed every 20 min of cooking, half of which was mixed with 55 wt % ground goethite powder. Lap-shear tests were then conducted with the pure bitumen and the bitumen and ochre samples of each cooking step (all values reported in table S2). The maximum strength of pure bitumen adhesives was very low (Fig. 4), with τ*_u_* < 0.01 MPa (i.e., no strength signal could be measured, and the laps slid apart with no measurable resistance; we therefore conducted only a single test with these samples). Cooking for longer produced no change in pure bitumen, i.e., all τ*_u_* values remained <0.01 MPa. Compound adhesives yielded strength values greater than those of pure bitumen (Fig. 4A). Cooking for 80 min and more resulted in a two- to threefold increase of strength in compound adhesives as compared to pure bitumen: Bitumen cooked for 80 min + 55 wt % ochre had τ*_u_* = 0.021 ± 0.01 MPa; bitumen cooked for 100 min + 55 wt % ochre had τ*_u_* = 0.022 ± 0.01 MPa (Fig. 4B), as determined from five consecutive tests with these samples. After 107 min of cooking, we had to stop the experiment because the mass hardened almost instantaneously, becoming a tough solid with no adhesive properties, which could not be used for bonding together laps. The overcooked bitumen had no adhesive properties even when we tried to soften it with a Bunsen burner. We conducted a second lap-shear experiment with air-dried bitumen. Pure air-dried bitumen yielded a significantly higher maximum strength with τ*_u_* = 1.49 +1.07 −0.81 (as determined by 10 tests). Mixing in 55 wt % goethite ochre reduced the air-dried bitumen’s maximum strength, resulting in τ*_u_* = 0.55 +0.53 −0.47 (as determined by nine tests; one test failed). Thus, making compound adhesives from air-dried bitumen and 55 wt % ochre has a negative effect, worsening the bitumen’s strength by a factor of 3 (Fig. 4C). When mixed with fresh bitumen, 55 wt % ochre increases its strength by a factor of 3. Nonetheless, our values measured on fresh bitumen are very low if compared to lap-shear data reported on other archaeological adhesives [which fall in the range of τ*_u_* = 0.1 to 1.5 MPa (*9*, *62*–*64*)]. We therefore attempted to identify other possible performance gains of a high ochre load in bitumen. It is known from Neanderthal birch tar finds (*5*, *32*) that adhesives were used as handles directly attached to stone tools (as opposed to being used for hafting stone tools to wooden handles or shafts). We therefore molded handles of fresh bitumen to stone tools (Fig. 4F, also see fig. S12). When manipulated, such pure bitumen grips are sticky to the touch and part of the bitumen is removed, leaving sticky stains on the hand, which are difficult to remove (Fig. 4E). When mixed in 55 wt % goethite ochre (Fig. 4G), bitumen grips feel more solid and are not sticky to the touch. When handling such a grip, no bitumen sticks to the manipulator’s hands (Fig. 4F). Thus, mixing high ochre loads in fresh bitumen presents an advantage for such composite tools. This type of tool use is supported by paleoanthropological data, as both Neanderthals and Middle Paleolithic *H. sapiens* are known to have habitually engaged in hand movements that involve precision gripping (*65*, *66*). These findings, and the observation that bright polishes and abrasion can be found across the colored part of the tools, make it most likely that the five stone tools were used in such adhesive grips rather than hafted to a rigid handle.

## DISCUSSION

The Le Moustier artifacts show that the European Middle Paleolithic adhesive technology was based on very similar processes as that in Africa. In both contexts, early humans deliberately produced some of their adhesives by distillation, from *Podocarpus* conifers in Africa (*9*) and birch bark in Europe (*21*, *22*). Both also mixed some of their glues with ochre to make compound adhesives (*14*, *67*). However, what do these behaviors actually imply in terms of cognition or cultural processes? Pioneering work on adhesive technology by Wadley *et al*. (*2*, *17*) suggests that compound adhesives have different implications than single-component [“simple” (*34*)] glues. This is because specific recipes must be followed, demanding multitasking and the use of abstraction and recursion (*18*). Others have approached the meaning of archaeological artifacts by quantitative interpretations of the steps needed for their production and their process complexity (*68*). There is a variety of methods for interpreting such stepwise complexity. In the case of adhesive technology, they range from graphical representations of compound adhesive production sequences (*69*) to computer-aided networks that correlate the steps and materials needed for adhesive making (*70*). Such approaches apparently allow comparisons among processes but depend on the scientist’s initial choice of steps. Because very different production pathways may produce the same finished products and because the actions of Pleistocene hominins do not fossilize, there is no mechanism to verify whether the chosen steps really took place. Therefore, we cannot decide whether the Le Moustier compound adhesives document more or less complex technology than other adhesives, such as birch tar. An alternative approach (*12*) proposes that adhesive making may have implications for human evolution if it relied on cultural transmission, was more difficult to execute, and/or was more costly than other techniques. In this sense, the Le Moustier adhesives document the willingness to invest more time and raw materials than other contemporaneous tool-making behaviors—i.e., their cost was likely higher. This is so because bitumen, flint, and pure goethite ochre do not occur at the same outcrops but must be gathered and transported to produce the composite tool. The Aquitaine basin, where the site of Le Moustier lies, contains abundant bitumen sources (*71*) and extensive flint outcrops (*72*), and goethite ochre is naturally available (*73*). However, the precise origin of the materials used for the Le Moustier multicomponent tools is not likely within close range of each other. The most prominent outcrop of pure goethite, the Grès de Thiviers (*73*), lies in a relatively close range to Le Moustier about 50 km to its north, but the closest Aquitaine oil fields, where bitumen outcrops can be found, lie more than 200 km to its south (*74*), roughly at equidistance to the petroleum sources of the Massif Central in central France. Thus, gathering the raw materials used for the Le Moustier compound adhesives was substantially more time- and effort-intensive than, for example, the collection of birch bark for tar making. In other words, compound adhesives were more expensive to their makers than adhesives like birch tar. The cost associated with these compound adhesives was higher than many other objects made in the European Middle Paleolithic, and their use likely implied cognitive processes, such as forward-planning and imagination (*18*), which were not required for many other Middle Paleolithic tools. Unfortunately, the context of Le Moustier allows reasonable doubts as to whether the authors of these pieces were Neanderthals. This is so because there are no radiometric dates available for our assemblage and direct dating of the lower shelter at Le Moustier [56 to 40 ka (*75*)], which is adjacent to the upper shelter from where our adhesives were excavated, situates the site at the end of the Neanderthal presence in Europe. At this time (*76*), and even before (*33*, *77*), *H. sapiens* incursions into southern Europe make it possible that Neanderthals and *H. sapiens* were present at the same sites.

So, what are the implications if these ochre-based compound adhesives were made by Middle Paleolithic *H. sapiens*? It is known from the African MSA record that very similar ochre-based compound adhesives were used (*67*). If anatomically modern humans brought this knowledge with them during their Out-of-Africa migration, its presence at Le Moustier would document a remarkably long technological continuity. In this case, the knowledge and skill to produce moldable materials with specific properties that are not available in single-component adhesives might have provided an advantage over the adhesive technology present in Europe at this time. Such a scenario would gain support from a recent proposal that contemporaneous *H. sapiens* at Mandrin Cave used a microlithic technology that relied on hafting (*78*). If future studies would find an association of the Le Moustier artifacts with early *H. sapiens* incursions [also see (*79*)], they might become an important argument in the debate surrounding the latter’s technological superiority over more archaic humans [see, for example, (*80*–*82*)].

Alternatively, what are the implications if these compound adhesives were made by Neanderthals? It has recently been shown that Neanderthal birch tar making relied on the cumulative cultural transmission of techniques (*32*). This was proposed to be one of the core criteria for understanding ancient adhesives as proxies of evolutionary processes (*12*). Another proposed core criterion is the willingness to invest elevated costs in the production of tools (*12*). If Neanderthals were the authors, the Le Moustier artifacts would constitute the second indication that their adhesive technology can be regarded as highly relevant for studying their cultural evolution. Another implication would result from ochre being used instead of other fillers. It is known that ochre has properties that lend it to its use in both utilitarian and symbolic contexts (*17*, *83*, *84*), and some authors argue that its use is never entirely utilitarian (*85*, *86*). Following that argument, if Neanderthals made the Le Moustier compound adhesives and they used the ochre for both its utilitarian and symbolic purposes, the evidence presented here would add weight to arguments in favor of the Neanderthal capacity for symbolic behavior (*87*–*89*). In any case, the use of ochre as filler would appear to be present in African *H. sapiens* and Neanderthals, both showing almost identical adhesive technologies in all aspects (the only difference being the types of raw materials used).

This obviously gives rise to the question: Is it possible that Neanderthals made the compound adhesives but that these similarities are the result of acculturation? This might be a likely scenario if contemporaneous *H. sapiens* in this region were known to produce compound adhesives from bitumen and ochre. There are adhesive finds associated with the late Aurignacian of the greater region (*23*, *24*), but these are of unknown composition and either are single-component (*23*) or contain no ochre [a quartz IR spectrum is presented as evidence of ochre in (*24*)]. They also postdate the Le Moustier assemblage. Thus, the still fragmentary adhesive find record does not allow us to decide whether acculturation is a likely scenario or not. The roughly contemporaneous find of one artifact containing an adhesive admixture based on coniferous resin and beeswax at Fossellone in Italy (*20*) most likely cannot shed light on this question either (the authorship of the Fossellone and Sant’Agostino adhesives may also be regarded as ambiguous; no ocher was reported; only 1 of 15 artifacts was proposed to be a compound adhesive; this was based on long-chain fatty acids and alcohols, and the typical wax esters were not reported).

In conclusion, the Berlin Le Moustier artifacts are the oldest compound adhesives that have been found in a European context. We found that high proportions of ochre make bitumen more rigid and prevent it from sticking to the hand. This suggests that the adhesives were used as handles directly attached to stone tools rather than for hafting stone tools to handles. This is in continuity with the behavior known from the European Middle Paleolithic (birch tar handles). Our findings also highlight the importance of the Middle Paleolithic adhesive technology for our understanding of the economy and technology of the foragers of this period. They invested time and effort in making compound adhesives and had the cognitive capacities needed. This technology provided new materials with specific and desirable material properties. This capacity and the willingness to invest in tools that represented an elevated cost documents the complexity of late Middle Paleolithic hominin behavior.

## METHODS

IR spectra were recorded from KBr pellets by direct transmission (in a vacuum chamber), using a Bruker VERTEX 80v spectrometer, spectral acquisition between 1800 and 400 cm^−1^, and a resolution of 2 cm^−1^. The ~0.3-g pellet contained 1 mg of sample.

CT scans were recorded with a Nikon XT H 320 CT scanner in Tübingen, selecting a resolution of about 11.5 μm (XrayA = 215 mA and ErayV = 215 kV). The reconstructed volumetric data (.vol) were sliced, and the ISO surface of the pieces was generated using the VGSTUDIO MAX version 3.5.1 software.

Lap-shear tests were performed in uniaxial tension and with a speed of 1 mm/min, using a universal testing machine (Instron 4502). All tests were performed at 21°C. Aluminum Laps (100 mm × 25.4 mm) were mounted in kardanic suspended tensile grips to minimize the bending moment on the samples. Contact zones (12.7 mm × 25.4 mm, 322.6 mm^2^) were abraded with 100 grit sand paper. Tests result in stress/strain diagrams, plotting apparent shear stress τ in megapascal (as obtained from the force applied by the testing machine/bonded area) over strain in percent.

## Supplementary Materials

### This PDF file includes:

Supplementary Text Figs. S1 to S12 Tables S1 and S2 References
