# Supplementary material for: Ochre-based compound adhesives at the Mousterian type-site document complex cognition and high investment
Source: Sci Adv. 2024 Feb 21;10(8):eadl0822. doi: 10.1126/sciadv.adl0822 (PMC10881035; doi:10.1126/sciadv.adl0822)
Supplement: Supplementary file 1 — Supplementary Text Figs. S1 to S12 Tables S1 and S2 References [file sciadv.adl0822_sm.pdf]

Science Advances
AAAS

Supplementary Materials for

Ochre-based compound adhesives at the Mousterian type-site document complex cognition and high investment

Patrick Schmidt *et al.*

Corresponding author: Patrick Schmidt, patrick.schmidt@uni-tuebingen.de

Sci. Adv. **10**, eadl0822 (2024)
DOI: 10.1126/sciadv.adl0822

This PDF file includes:

Supplementary Text
Figs. S1 to S12
Tables S1 and S2
References

Supplementary text

The origin of the artefacts

The research history of the Le Moustier artefacts studied here spans over more than 100 years. They are now curated in the "Tode collection" at the Museum für Vor- und Frühgeschichte in Berlin (Germany). Their origin can be traced back to the excavations of Swiss archaeologist Otto Hauser (1874-1932) who unearthed the pieces during his works in the Dordogne region (southwestern France) in the beginning of the 20th century. The most likely date of excavation is Hauser's field campaigns at the upper rock shelter of Le Moustier in August 1907. This upper rock shelter, also called *abri classique*, *abri supérieur* or Station 43, was discovered by pioneer archaeologists Eduard Lartet and Henry Christy in 1863. The lithic artefacts from the site gave rise to its nomination as the eponymous site of the "Mousterian" (the name was proposed by Gabriel de Mortillet) (*89-94*). The first stratigraphic profiles of the upper terrace at Le Moustier (containing Mousterian industries at its bottom and Aurignacian artefacts on top) were published by Maurice Bourlon and Denis Peyrony (*95-100*). In 1963, the Museum für Vor- und Frühgeschichte in Berlin acquired 100 artefacts from Hauser's 1907 excavation from the Väterkunde Museum in Bremen (Germany, today Ludwig-Roselius-Museum für Frühgeschichte) (File-No. MVF 0000/1504) (*102*), where Alfred Tode (1900-1996) was the director at the time. Ninety-seven (*96*) of these artefacts are currently curated in the "Tode collection" at the Museum für Vor- und Frühgeschichte in Berlin. The museum records mention the pieces as (translated) "*The best selected pieces of the typical Mousterian […] from the famous site of Le Moustier, upper terrace*". They were acquired for the museum's collections along with a greater collection of artefacts under the number EB1963:30 (File-No. MVF 0000/1504) (*102*). This description of the origin of the Berlin artefacts allows for their assignment to the *abri classique* of Le Moustier. Investigations made by Almut Hoffmann (*103*) (based on oral communications with successor institutions in Bremen and State Archaeologist Ralf Busch) indicate that, after the death of Otto Hauser in 1932, his widow Erna Hauser sold artefacts to various institutions in Braunschweig, Bremen, Hamburg and Wolfenbüttel (*101, 103*). Thus, the artefacts can be reasonably well traced back to the Mousterian layers excavated by Hauser in 1907 at Le Moustier.

Description of the Tode collection

The lithic artefacts of the Tode collection include blanks typologically classified as "Levallois", such as preferential flakes, points and blades (*35*). The retouched inventory includes scrapers with one or two retouched edges, as well as denticulated pieces and oval-shaped, bifacial handaxes. From a typological viewpoint, no occurrences of a standardized blade or bladelet blank production and no technological indications, which would point towards indirect soft-hammer percussion, are recorded. The absence of end-scrapers, backed knives, and Châtelperronian points excludes the assignment of the assemblage to a transitional or an Upper Paleolithic industry.

Therefore, and despite the selective character resulting from the early excavation of the assemblage, our techno-typological observations are qualitatively reminiscent of the Mousterian. It is highly likely that the assemblage allows an assignment of the artefacts to the Mousterian (*105*), as originally noted in the acquisition documents. This argues for a clear attribution of the assemblage to the Middle Paleolithic.

List of artefacts, micro-wear traces, and descriptions

Table S1. List of artefacts analyzed in this study.

Accession n°	Typology	Ochre	Bitumen	Use-wear
Va 7157.6	End-notched flake	yes	yes	Polish on distal part
Va 7136	Levallois flake	yes	yes	Polish on distal part
Va 7157.9	Retouched flake	yes	yes	Polish on distal part
Va 7158.7	Retouched Levallois blade	yes	yes	Polish on distal part
Va 7157.24	Side scraper	yes	no	Not tested, patinated

Methods of our preliminary use-wear analysis

The five artefacts were investigated with a Leitz Orthoplan metallographic (reflected light) microscope, set up in co-axial illumination (bright-field). The objective (16x) had a numerical aperture of 0.3 and resulted in a 200x magnification with the eye pieces (12.5x). We inspected both faces of each artefact including all edges in search for use- and hafting-related traces (*36, 38, 105, 106*), including striations (*106, 107*), polish (*108*), and micro-fractures (*109*). The artefacts were cleaned with acetone and isopropanol at their edges to remove traces of dust, finger grease, and varnish. A thorough cleaning with modern methods, such as using ultrasonic baths, could not be performed because of the importance of preserving the residues. Nonetheless, the pieces do not contain traces of sediment and we assume that they were previously cleaned. We present only the most obvious traces relevant to establishing that the tools were used and held in an adhesive haft/grip. We refrain from a full functional interpretation of the tools, which will be the object of a dedicated study performed on the entire collection.

Descriptions of the artefacts

Va 7157.6 is a 6.97 cm long, 5.38 cm wide and 1.46 cm thick (47.4 g) end-notched flake. A drawing showing the distribution of colorant stains and micrographs showing use wear traces are shown in Fig. S1. The tool contains a cortical part at its proximal right side, forming a natural back. The adhesive protuberance analyzed her is preserved on this natural back. The flake is retouched into a notch on its left distal side and forms a point. There is polish on the ventral face, indicating that it was used. It is not typically a borer, lacking striations perpendicular/transversal to the active edge. Red colorant forms a band across the ventral and dorsal faces. Black residue is preserved associated with the colorant stains. There is invasive polish on the ventral face of the presumed active edge. Large parts of the proximal portion of the tool show bright polish and striations beneath red and yellow colorant stains. Such polish is absent from the presumed active portion of the tool (which we assume was not held in an adhesive grip). The association between adhesive remains and polish on the surface of the tool, i.e. away from the working edge, suggests that the stone tool was abraded against an ochre containing adhesive, perhaps in a grip that still allowed movement of the stone tool.

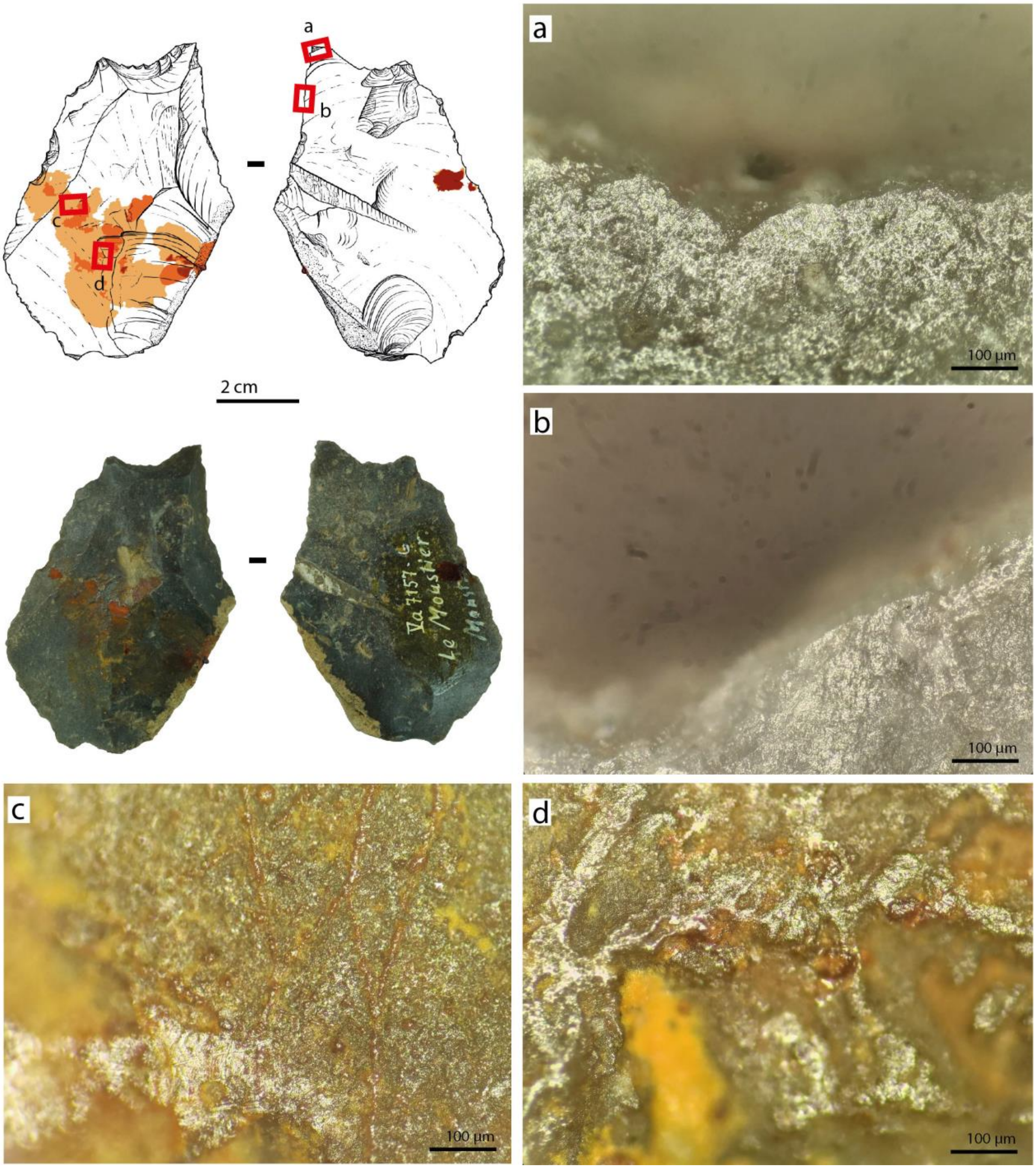

Fig. S1. Micrographs showing wear traces on artefact n° Va 7157.6. Locations of the micrographs on the artefact are indicated in the drawing (upper left) in red. a, b) Polish on active edge. c, d) Polish and striations under colorant stains within the zone covered by adhesive. Drawing by D. Greinert, Staatliche Museen zu Berlin.

Va 7136 is a 7.76 cm long, 4.11 cm wide and 1.47 cm thick (31 g) unretouched Levallois flake. A drawing showing the distribution of colorant stains and micrographs showing use wear polish are shown in Fig. S2. The tool contains irregular use-related bifacial micro-fractures on its presumed active edge. A mixture of red and yellow colorant and black adhesive is preserved on the right edge, on both faces. The edges of the base, presumably situated in the adhesive grip, show little to no micro-fractures. There is invasive polish on both sides of the ventral face of the presumed active edge.

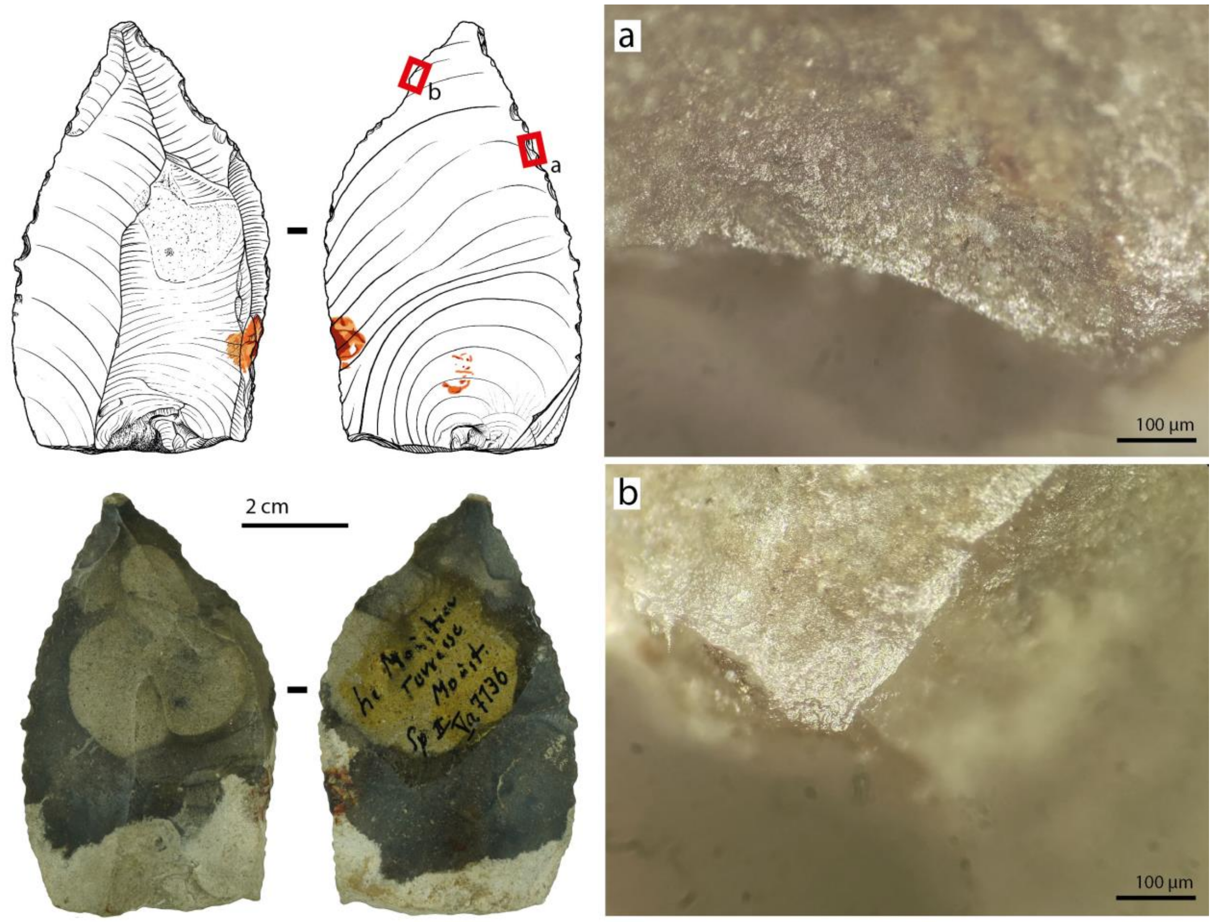

Fig. S2. Micrographs showing wear traces on artefact n° Va 7136. Locations of the micrographs on the artefact are indicated in the drawing (upper left) in red. a, b) Polish on active edge. Drawing by D. Greinert, Staatliche Museen zu Berlin.

Va 7157.9 is a 6.02 cm long, 4.56 cm wide and 1.4 cm thick (24.4 g) retouched flake. A drawing showing the distribution of colorant stains and micrographs showing use wear traces are shown in Fig. S3. Retouch can be found at the distal right side on the dorsal face. A band of red and yellow colorant is associated with black adhesive residues. The edges show damage on the right side and the distal part of the left side of the artefact. There is polish on the ventral face at the retouch, the presumed active edge of the artefact (which was not covered by the adhesive). Within the presumed passive part of the tool, i.e. the portion covered in adhesive, there is bright polish that is absent on the presumed active part of the tool. This polish appears to abrade the ridge between negatives on the dorsal side. The strict association of such polish with the zone within the portion covered by adhesive, as supported by

the association of polish and colorant stains, indicates abrasion of the flint against an ochre-containing adhesive grip during utilization of the composite tool.

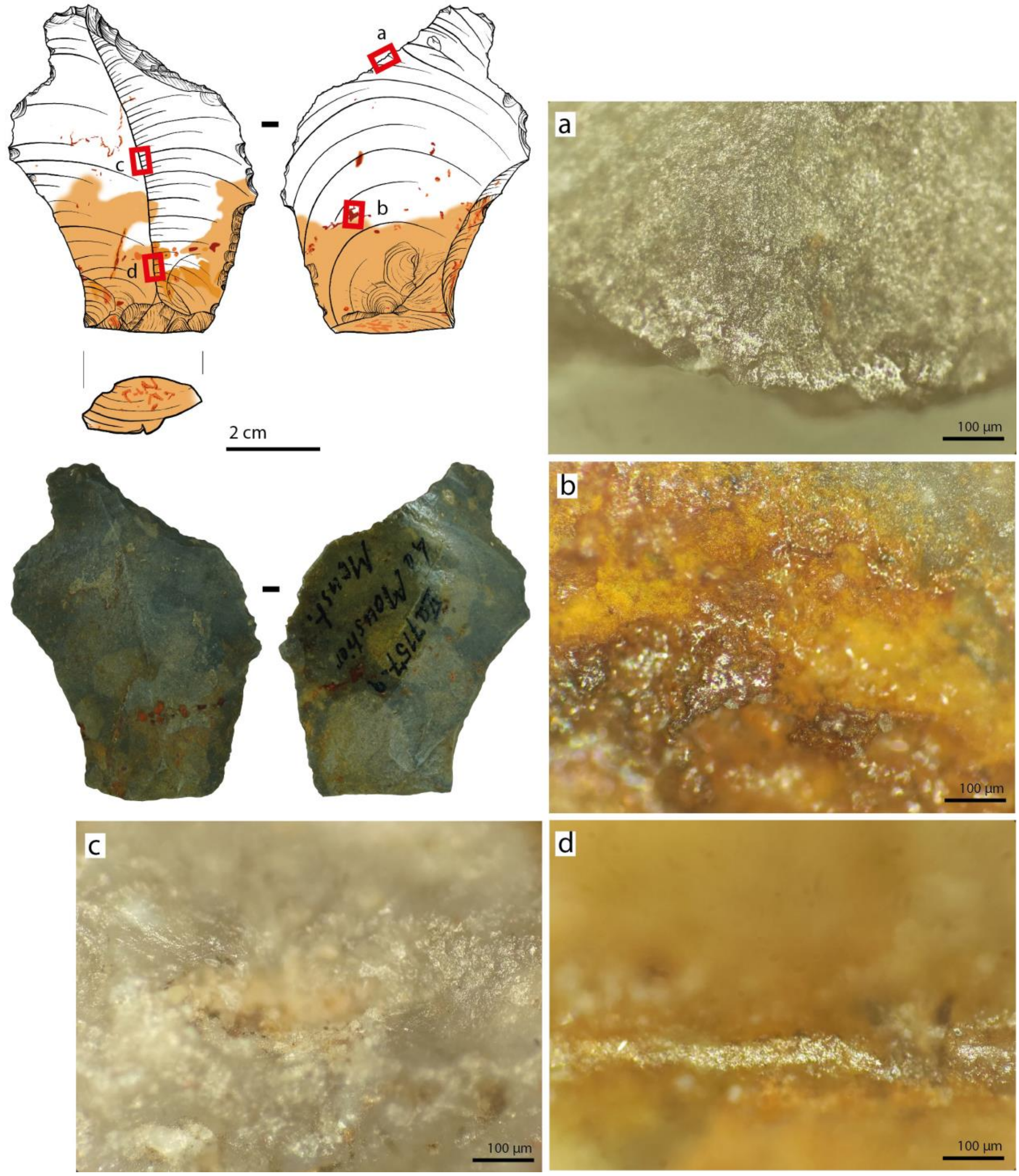

Fig. S3. **Micrographs showing wear traces on artefact n° Va 7157.9.** Locations of the micrographs on the artefact are indicated in the drawing (upper left) in red. a) Polish on active edge. b) Polish under colorant stains within the zone covered by adhesive. c) Ridge between removal negatives that is not dulled out or abraded. d) Dulled out (abraded) ridge in the prehensile zone that was covered in adhesive. Compare micrographs (c) and (d). Drawing by D. Greinert, Staatliche Museen zu Berlin.

Va 7157.24 is an 8.7 cm long, 4.98 cm wide and 1.28 cm thick (54.5 g) side scraper. A drawing showing the distribution of colorant stains and a micrograph showing use wear polish are shown in Fig. S4. The artefact has a cortical right side, forming a natural back. Its left side is retouched from the proximal part to the distal part. Yellow colorants are preserved on the dorsal side. The piece is lightly patinated (white dissolution patina). We therefore did not analyze use-wear on its edges. There is bright polish associated with colorant stains on the dorsal side. Such polish is absent from the presumed active part that was not covered by the adhesive.

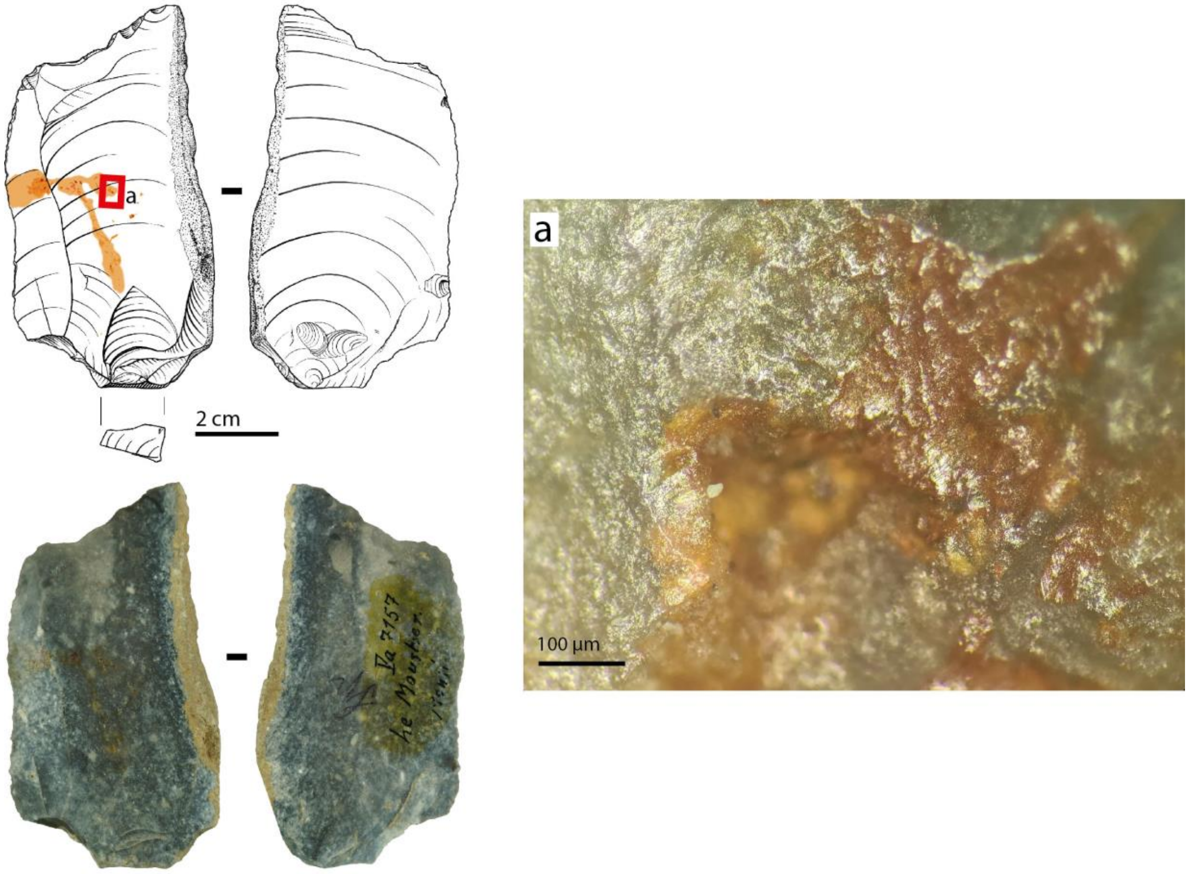

Fig. S4. **Micrographs showing wear traces on artefact n° Va 7157.24.** The location of the micrograph on the artefact is indicated in the drawing (upper left) in red. a) Polish under colorant stains in the zone covered by adhesive. Drawing by D. Greinert, Staatliche Museen zu Berlin.

Va 7158.7 is a 7.56 cm long, 3.91 cm wide and 1.19 cm thick (40.3 g) retouched Levallois blade. A drawing showing the distribution of colorant stains and micrographs showing use wear traces are shown in Fig. S5. The artefact is retouched on its left side. Retouch on its right side is only present at the presumed active portion of the artefact (the part not held in the adhesive grip). The platform preserves a cortical plan. Spots of red colorant are present on both faces and are associated with black residue. One reddish to yellowish colorant spot is located on the dorsal face of the presumed active portion of the tool. Below the colorant stains, there is bright polish indicating abrasion of the flint surface against the adhesive grip. In the portion covered by the adhesive (the proximal part of the blade), ridges between removal negative are dulled out. This indicates that the entire proximal portion

of the blade was covered by the adhesive grip. Such polish or dulling of ridges between removal negatives is absent in the presumed active portion of the tool.

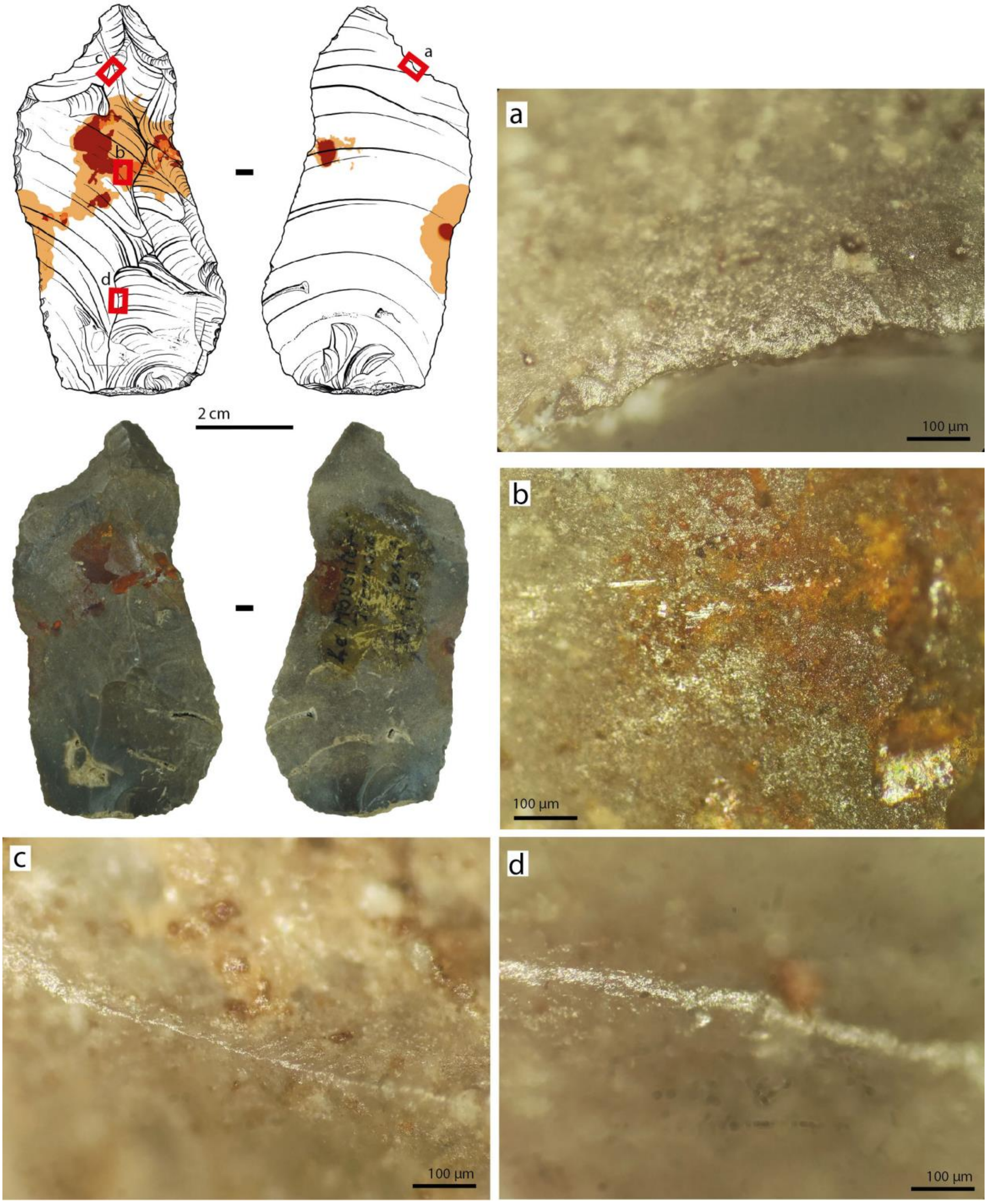

Fig. S5. **Micrographs showing wear traces on artefact n° Va 7158.7.** Locations of the micrographs on the artefact are indicated in the drawing (upper left) in red. a, b) Polish and striations under colorant stains within the zone covered by adhesive. c) Ridge between removal negatives that is not dulled out or abraded. d) Dulled out (abraded) ridge in the prehensile zone that was covered in adhesive. Compare micrographs (c) and (d).

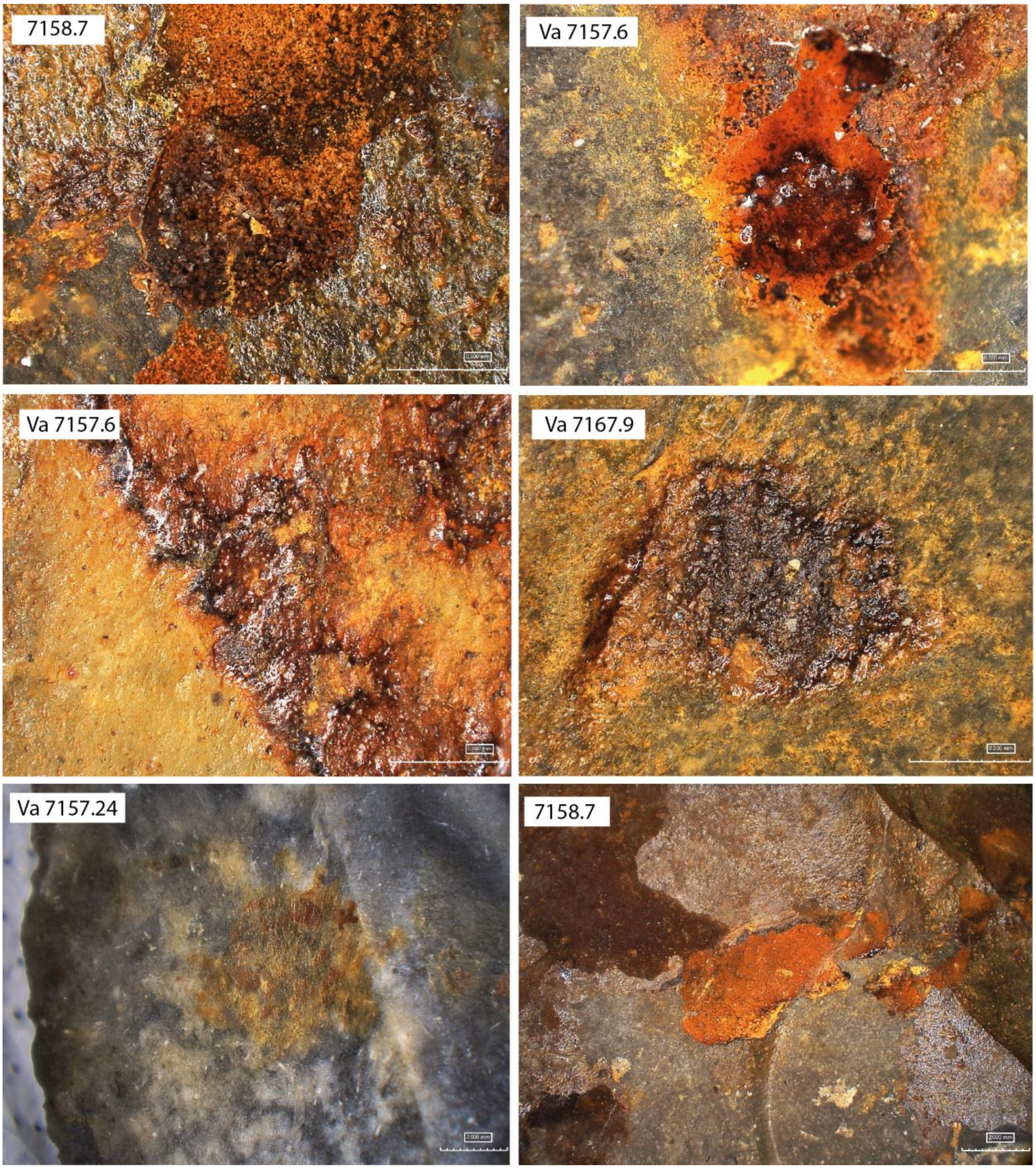

Fig. S6. **Micrographs showing colorant stains and the association of colorant with black adhesive on the artefacts.**

Supplementary data on our IR analysis

Sample preparation and instrument

Infrared spectra of the Le Moustier residue and references samples of conifer resins, birch tar, bitumen and goethite were recorded from KBr pellets by direct transmission, using a Bruker VERTEX 80v spectrometer, spectral acquisition between 1800 cm^{-1} and 400 cm^{-1} and a resolution of 2 cm^{-1}. During analysis, samples were held in a low-pressure chamber at < 3 hPa. Each ~ 0.3 g pellet of reference samples contained 0.7 mg of sample. The pellet of the Le Moustier sample contained 1 mg of sample.

Comparison with other potential naturally available adhesives

To exclude that the organic fraction of the Le Moustier residue is of botanical origin, we compare its spectrum with that of resins of spruce (*Picea abies*), fir (*Abies alba*) and pine (*Pinus pinea*) and with birch tar made from *Betula pendula* (Fig. S7). The four references all show a strong band caused by C=O. The band is absent in the Le Moustier spectrum. The Le Moustier spectrum shows a S=O band characteristic of crude oil and bitumen. The comparison rules out that the Le Moustier residue is made of resin or birch tar. Note that there is a band at 1040 cm^{-1} in the spectrum of fir resin (*Abies alba*) and birch tar (there at 1033 cm^{-1}), which is caused by O-H in C-OH functional groups, most likely in cyclic diterpenoid molecules. Its wavenumber differs sufficiently from that of the S=O vibration in bitumen, that confusion between these substances can be comfortably ruled out. Also, note that there is a band at 1630 cm^{-1}, which is caused by water (an O-H bending vibration of the H_2O molecule). This band partly masks the bitumen spectrum. However, the position of C=O bands in tree resins and birch tar, near 1700 cm^{-1}, lies at sufficiently greater wavenumbers so that, if a C=O band were present in the Le Moustier spectrum, it could be recognized on the flank of the H_2O band.

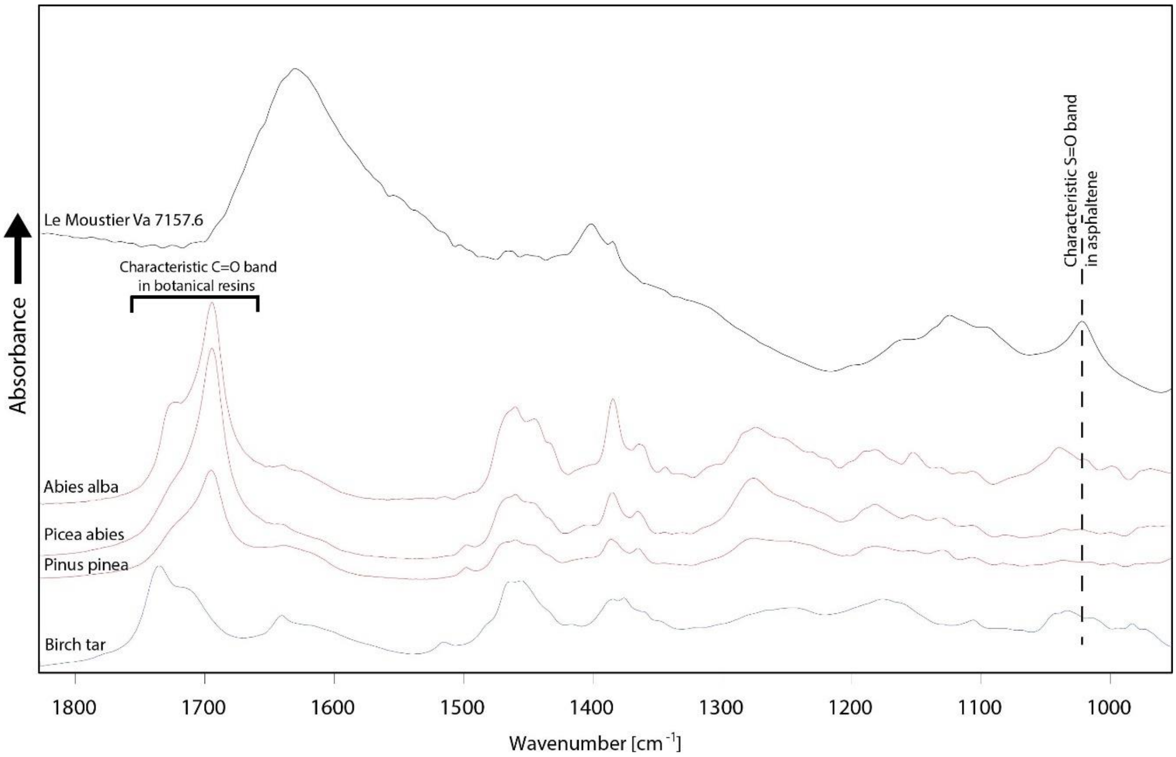

Fig. S7. Comparison of the organic bands in the fingerprint region of the Le Moustier spectrum with reference substances. Reference spectra are resins of spruce (*Picea abies*), fir (*Abies alba*) and pine (*Pinus pinea*) and birch tar made from *Betula pendula*. Note that the four botanical reference substances al have a strong C=O band absent in the Le Moustier spectrum. Also note that none of the four reference substances present a S=O band characteristic for bitumen and crude oil.

Supplementary data on our GC-MS analysis

To corroborate our finding that the Le Moustier residue was made from bitumen, we attempted to conduct Gas Chromatography-Mass spectrometry (GC-MS) analysis on the soluble fraction of the 1 mg

sample used for infrared spectroscopy (extraction of the KBr pellet using a dichloromethane/methanol mixture).

In GC-MS, crude oil and bitumen are normally identified by the presence of hopanes (*m/z* 191) and steranes (*m/z* 217 and 218), both having a characteristic saturated hydrocarbon skeleton. First, the extracted soluble part of the sample was injected in GC-MS without silylation, attempting to find these typical petrol biomarkers. Analysis by Single Ions Monitoring (SIM) on the fragments *m/z* 191, 217 and 218 did not yield signals of hopane or sterane. Then, the soluble part was analysed again after silylation, in order to search for any oxygenated polyterpenes characteristic of plant products (for example, diterpenic or resinic acids of conifer tars, triterpenic compounds of birch bark tar). The resulting chromatogram shows several fatty acids and alcohols with sitosterol, most likely resulting from contamination of the sample. We could exclude the presence of plant-derived di- or triterpenoids that would have been characteristic of natural resins or their tars. The most parsimonious interpretation of our GC-MS analysis is therefore that the low sample mass did not yield a sufficiently large soluble quantity above the detection limit of the method. This interpretation is strengthened by our infrared spectroscopic analysis that showed asphaltene. Altered bitumen contains a saturated hydrocarbon fraction potentially visible by GC-MS of less than 3 % (weight% of the organic extract) (*51*). The low proportion of hopanes and steranes in bitumen has also been observed by other authors, who mention ratios as low as 10 µg /g of the total organic carbon (*50*). If the residue consisted of plant-derived products (resins or their tars), its major part were soluble so that its chromatogram would likely have yielded evidence of characteristic terpenoids.

Sample preparation

The KBr pellet used for IR analysis, containing 1 mg of the Le Moustier adhesive sample, was first ground in an agate mortar. An organic extract was prepared by ultrasonic-assisted extraction three times by means of a mixture of dichloromethane/methanol 60:40 v/v (500 µl per extraction step). After concentration under gentle nitrogen flow, the organic extract obtained was filtered through diatomaceous earth to remove any insoluble residue (elution with dichloromethane/methanol 60:40 v/v) and again concentrated to dryness. After the initial GC-MS analysis without silylation, the remaining sample was further processed. For this, the injected fraction was concentrated under nitrogen flow and then engaged in the trimethylsilylation reaction. After the addition of 40 µl pyridine and 200 µl N,O-bis(trimethylsilyl)trifluoroacetamide (BSTFA), the reaction medium was heated for 2 hours at 70°C and then evaporated to dryness before being injected in GC-MS.

Instrument and GC-MS analysis parameters

The unsilylated and silylated organic extracts were dissolved in 10µl dichloromethane before being injected (2µl injected). GC-MS analyses were performed with an Agilent 8890 chromatograph coupled with an Agilent 5977B MSD. The temperature of the source was set at 220°C. The mass spectrometer was operating in electron impact (EI) mode at 70eV. Gas chromatographic separations were operating on a HP-5MS column (30m x 0.25mm x 0.25µm film thickness) with constant He flow of 1.5 mL/min and a temperature gradient of 40°C for 2min, then 10°C/min until 100°C, then 4°C/min up to 320°C, hold time for 60min. GC-MS interface was set at 320°C. Mass spectra were produced in full detection mode over 70-800 amu. Analysis in Single Ions Monitoring (SIM) mode was performed by selectively searching *m/z* 191, 217 and 218. Before each injection of the archaeological sample, two blanks are performed (injection of 2µl dichloromethane under the same conditions as the sample). Peak

assignment was based on the interpretation of mass spectra obtained with the OpenLab software and comparison with spectra available in the literature and NIST library 2.0.

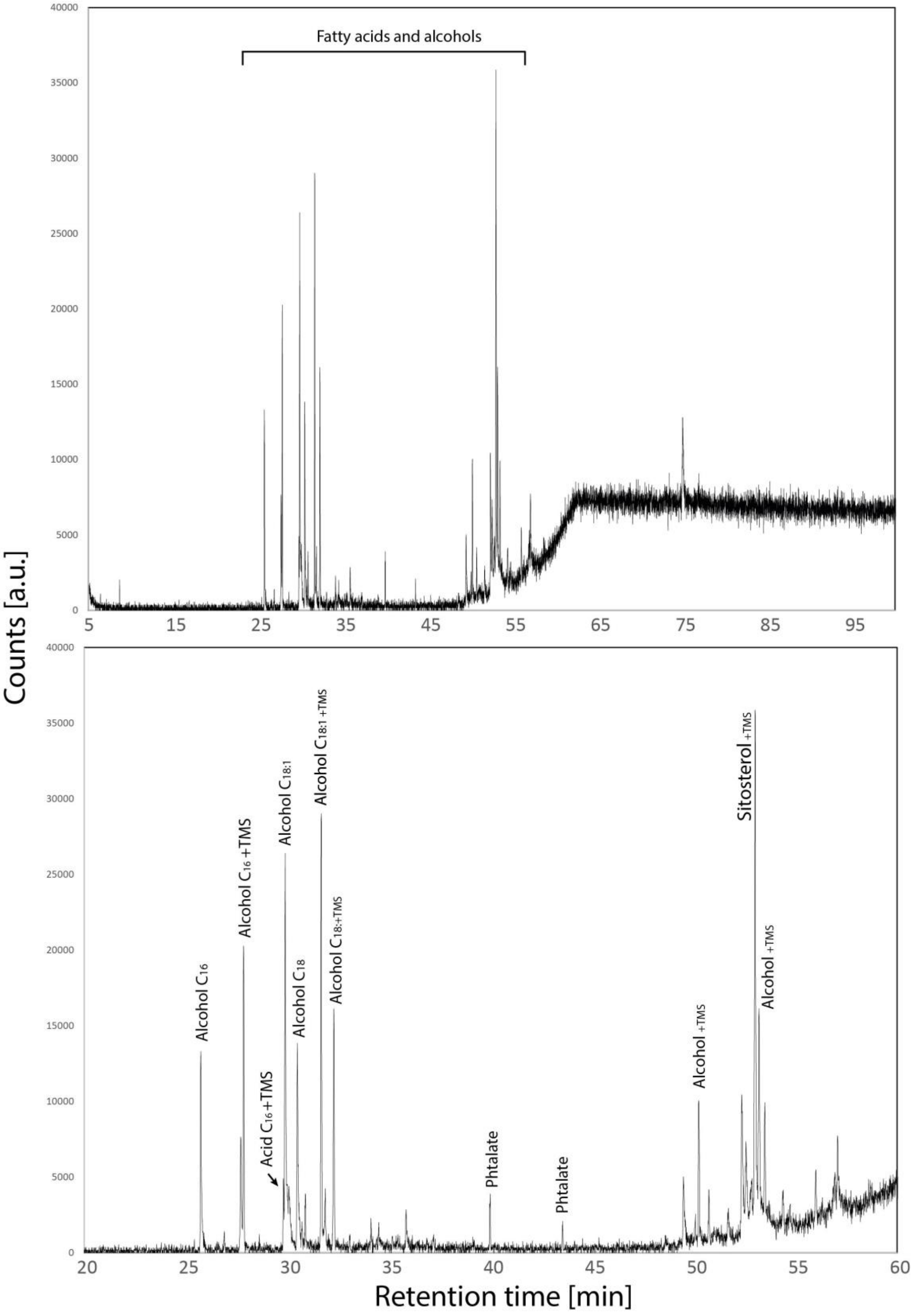

Fig. S8. **Full Chromatogram of the soluble fraction of artefacts n° Va 7157.6 after silylation (top) and crop between 20 and 60 min (bottom).** Note that only fatty alcohols and acids are found.

Chemicals used for our GC-MS analysis

Dichloromethane (Fisher Scientific) and methanol (Carlo Erba) were HPLC grade and were used without further purification. Pyridine, BSTFA and diatomaceous earth (Celite® 545) were purchased from Sigma Aldrich. Only dichloromethane cleaned glassware and, above all, no plastic material was used to avoid any contamination.

Supplementary data on our EDX analysis

Our EDX Analysis of the adhesive mass that is held together by the bitumen binder, as removed from artefact n° Va 7157.6, can be seen in Fig. S9.

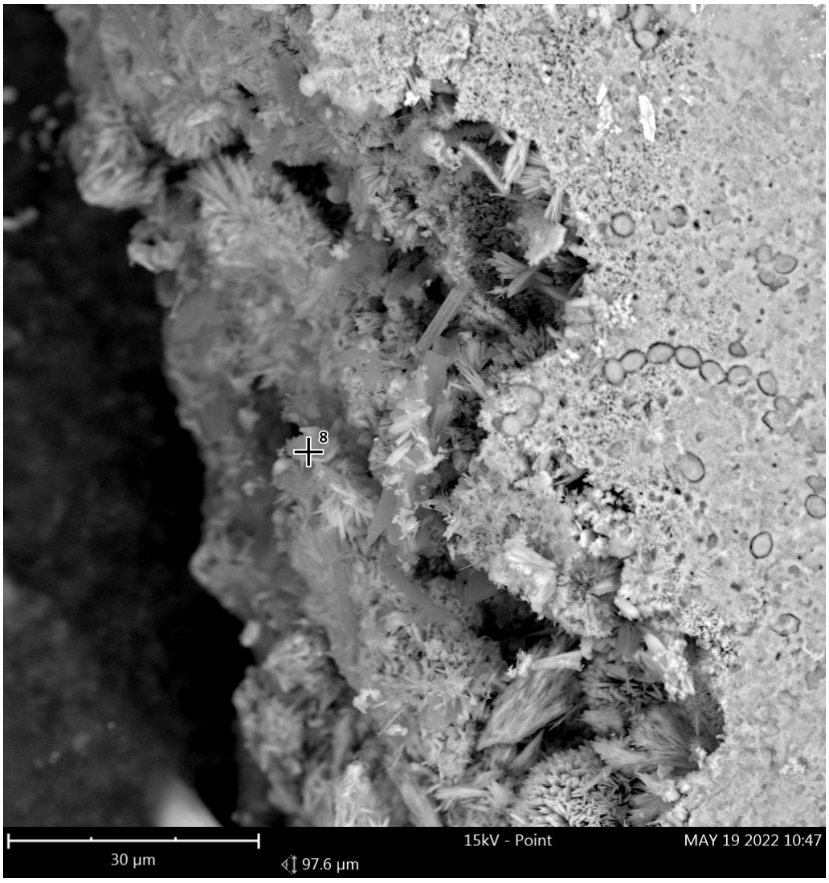

Element Symbol	Atomic Conc.	Weight Conc.
C	56.00	41.74
O	35.49	35.23
Fe	3.20	11.09
Ca	2.79	6.93
S	2.33	4.64

Fig. S9. **Detail showing the spot (as black +) measured by EDX on the Le Moustier adhesive removed from artefact n° Va 7157.6.** Mode: 15kV - Point, Detector: BSD Full.

The goethite ochre filler can be seen in detail in Fig. S10.

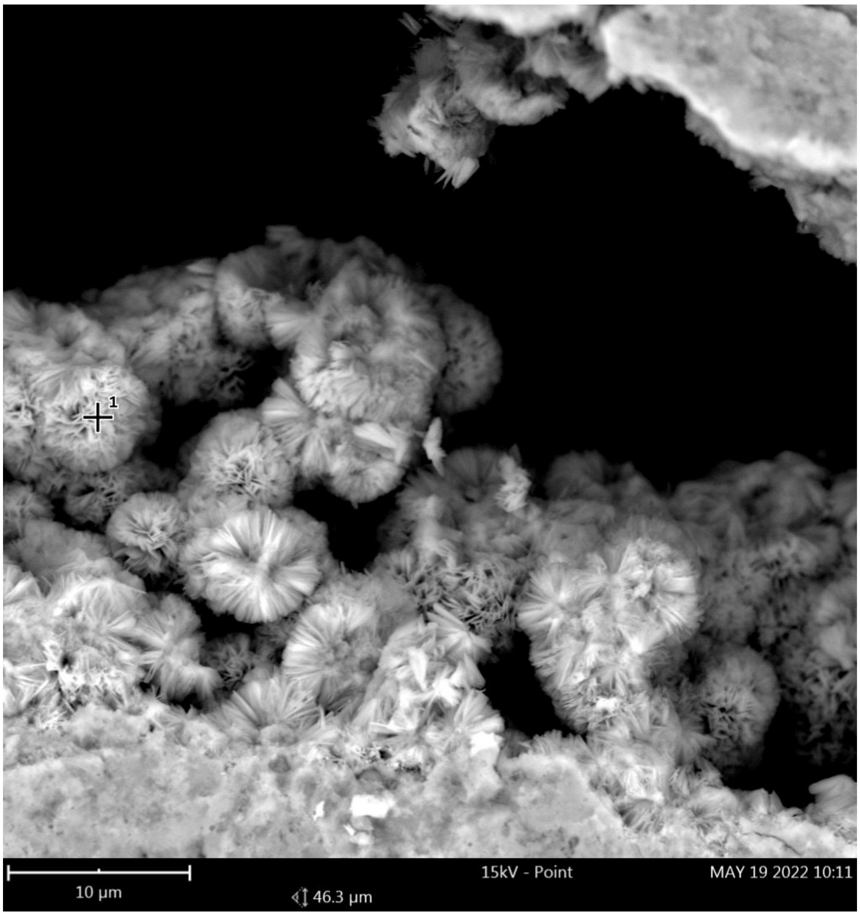

Element Symbol	Atomic Conc.	Weight Conc.
O	58.86	29.07
Fe	41.14	70.93

Fig. S10. **Detail showing the spot (as black +) measured by EDX on the goethite ochre filler of the Le Moustier adhesive removed from artefact n° Va 7157.6.** Mode: 15kV - Point, Detector: BSD Full.

Supplementary data on bitumen cooking and lap-shear tests

During bitumen cooking, we removed a sample after each 20 min of cooking time. Half of these samples were mixed with 55 wt% goethite ochre while still being warm. The ochre was therefore ground to obtain a fine powder in which individual grains formed no aggregates. Air-dried bitumen was gently heated on a hot plate (60°C) until it became liquid and then the ochre powder was mixed in to obtain a homogeneous mass. All samples (fresh and air-dried bitumen with and without ochre) were left to cool to room temperature before they were all plied to aluminum laps used for lap-shear tests by reheating them on the hot plat. Bonded laps were left to cool to room temperature before lap-shear testing began.

Lap-shear tests were performed in uniaxial tension and with a speed of 1 mm/min, using a universal testing machine (Instron 4502). All tests were performed at 21°C. Aluminum laps (100 x 25.4 mm) were mounted in kardanic suspended tensile grips to minimize the bending moment on the samples. Contact zones (12.7 x 25.4 mm, 322.6 mm^2) were abraded with 100 grit sand paper. The thicknesses of the adhesive bonds were measured as the total thickness of the two bonded laps, subtracting the laps' thickness. These tests result in stress/strain diagrams, plotting apparent shear stress τ in MPa (as obtained from the force applied by the testing machine/bonded area) over strain in percent.

As proposed by Schmidt et al. (*63*) we extracted two values from these stress/strain diagrams: maximum shear stress τ_u in MPa (the maximum stress value as read on the diagrams) and shear strength $\tau_{(yield)}$ in MPa (the stress at which the curve deviates from the tangent slope of the linear increase of τ). We also calculated shear modulus *G* (in GPa) as the slope of this tangent line in a slightly modified stress/strain diagram (reporting deformation strain, as Δ/d, where Δ is the elongation of the bonded laps as measured by the testing machine and *d* the thickness of the adhesive bond (*61*). Data are shown in Table S2.

Table S2. **Values obtained by lap-shear testing of pure bitumen and bitumen intermixed with 55% goethite ochre.**

	Lap n°	Shear Modulus *G* [GPa]	Shear strength $\tau_{(yield)}$ [MPa]	Maximum shear stress τ_u [MPa]
Air-dried bitumen	1	0.85	0.38	0.68
	2	1.17	0.73	2.08
	3	0.43	0.92	2.55
	4	0.63	0.49	0.91
	5	0.68	1.07	2.04
	6	0.29	0.90	1.6
	7	0.64	0.54	1.02
	8	0.42	0.86	1.3
	9	0.82	0.71	1.08
	10	0.59	0.87	1.59
Means		0.65 +0.52 -0.36	0.75 +0.32 -0.37	1.49 +1.07 -0.81
Air-dried bitumen + 55% Ochre	1	0.27	0.45	0.45
	2	0.45	0.46	0.46
	3	0.4	0.25	0.29
	4	0.36	0.42	0.84
	5	0.55	0.6	0.81
	6	0.57	0.07	0.08
	7	0.62	0.19	0.2
	8	0.19	1.06	1.08
	9	0.23	0.35	0.78
Means		0.4 +0.22 -0.21	0.43 +0.63 -0.36	0.55 +0.53 -0.47
Unheated fresh bitumen	-	-	-	0.00
Bitumen + 55% ochre	-	-	-	0.01
20 min Coked 20 min	-	-	-	0.00
+ 55% ochre	-	-	-	0.01
Coked 40 min	-	-	-	0.00
40 min + 55% ochre	-	-	-	0.01
Coked 60 min	-	-	-	0.00
60 min + 55% ochre	-	-	-	0.01
Coked 80 min	-	-	-	0.00

80 min + 55% ochre	1	-	-	0.03
80 min + 55% ochre	2	-	-	0.02
80 min + 55% ochre	3	-	-	0.01
80 min + 55% ochre	4	-	-	0.02
80 min + 55% ochre	5	-	-	0.02
			Mean:	0.021 ±0.01
Coked 100 min	1	-	-	0.00
Coked 100 min	2	-	-	0.01
Coked 100 min	3	-	-	0.01
Coked 100 min	4	-	-	0.01
Coked 100 min	5	-	-	0.01
			Mean:	0.006 +0.002 -0.001
100 min + 55% ochre	1	-	-	0.03
100 min + 55% ochre	2	-	-	0.02
100 min + 55% ochre	3	-	-	0.02
100 min + 55% ochre	4	-	-	0.01
100 min + 55% ochre	5	-	-	0.03
			Mean:	0.022 ±0.01

Supplementary information on bitumen sampling

Natural bitumen can be collected in bituminous lakes as a viscous liquid or as hardened air-dried mass on rock walls from where it seeps out. We sampled both types of outcrops.

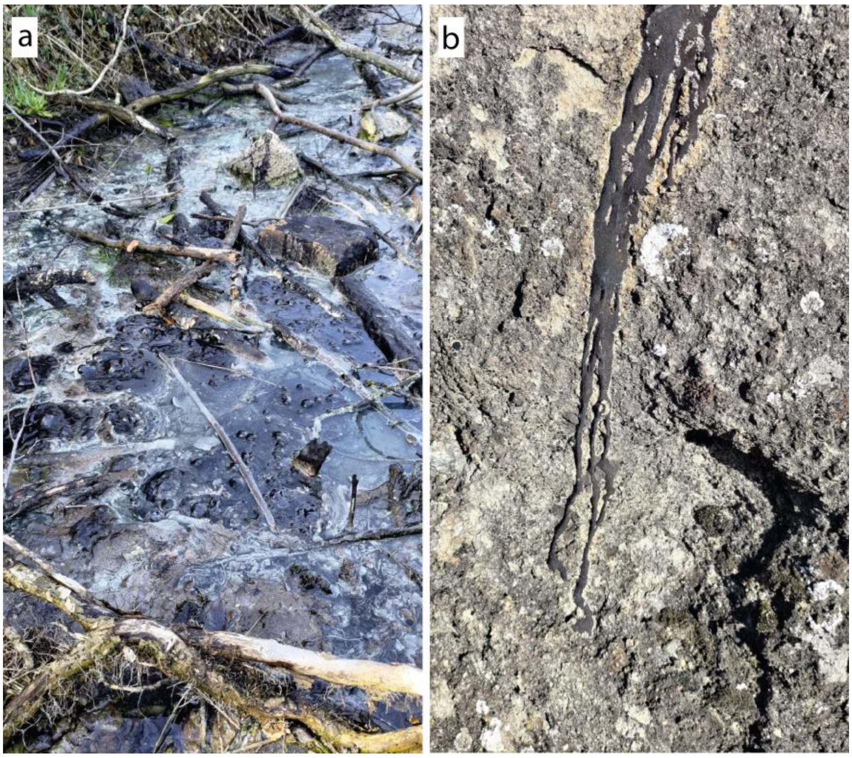

Fig. S11. Photos of the two sampled bitumen outcrops in the Massif Central (France). a) Bituminous lake with liquid fresh bitumen mixed with meteoric water. b) Air-dried bitumen seeping out of a rock cliff.

Hypothetical reconstruction of a handle made from the compound adhesive

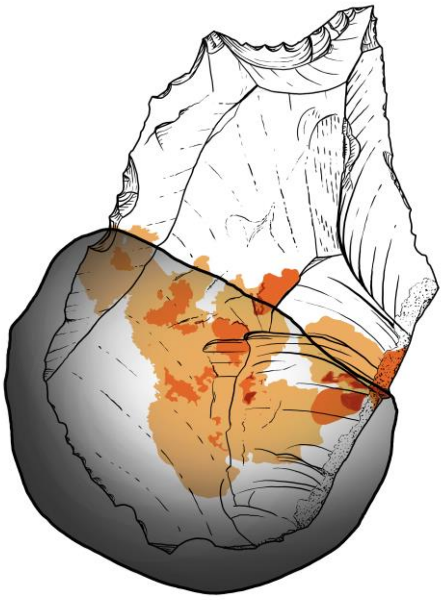

Fig. S12. **Hypothetical grip made of the Le Moustier compound adhesive on artifact n° Va 7157.6.**

Provenance

1) Where, when, and by whom the artifacts were collected

The artefacts analyzed in this study were excavated at the site Le Moustier (France) in 1907 by archaeologists Otto Hauser. In 1963, they were acquired by the Museum für Vor- und Frühgeschichte in Berlin (acquired under the number EB1963:30, File-No. MVF 0000/1504).

2) How, and by whom, the artifacts were validated for authenticity and aged/dated

Validation for authenticity and age was done by the Museum für Vor- und Frühgeschichte in Berlin, based on original files and descriptions, along with modern typological criteria.

3) Where the artifacts will be housed and/or how they can be freely accessed

The artifacts are currently, and will be, curated in the "Tode collection" at the Museum für Vor- und Frühgeschichte in Berlin. They can be accessed upon request at the museum.

REFERENCES AND NOTES

1. S. McBrearty, A. S. Brooks, The revolution that wasn't: A new interpretation of the origin of modern human behavior. *J. Hum. Evol.* **39**, 453–563 (2000).

2. L. Wadley, T. Hodgskiss, M. Grant, Implications for complex cognition from the hafting of tools with compound adhesives in the Middle Stone Age, South Africa. *Proc. Natl. Acad. Sci. U.S.A.* **106**, 9590–9594 (2009).

3. L. Backwell, F. d'Errico, L. Wadley, Middle Stone Age bone tools from the Howiesons Poort layers, Sibudu Cave, South Africa. *J. Archaeol. Sci.* **35**, 1566–1580 (2008).

4. T. Minichillo, Raw material use and behavioral modernity: Howiesons Poort lithic foraging strategies. *J. Hum. Evol.* **50**, 359–364 (2006).

5. M. J. L. T. Niekus, P. R. B. Kozowyk, G. H. J. Langejans, D. Ngan-Tillard, H. van Keulen, J. van der Plicht, K. M. Cohen, W. van Wingerden, B. van Os, B. I. Smit, L. W. S. W. Amkreutz, L. Johansen, A. Verbaas, G. L. Dusseldorp, Middle Paleolithic complex technology and a Neandertal tar-backed tool from the Dutch North Sea. *Proc. Natl. Acad. Sci. U.S.A.* **116**, 22081–22087 (2019).

6. K. S. Brown, C. W. Marean, A. I. R. Herries, Z. Jacobs, C. Tribolo, D. Braun, D. L. Roberts, M. C. Meyer, J. Bernatchez, Fire as an engineering tool of early modern humans. *Science* **325**, 859–862 (2009).

7. T. Wynn, Hafted spears and the archaeology of mind. *Proc. Natl. Acad. Sci. U.S.A.* **106**, 9544–9545 (2009).

8. V. Rots, P. Van Peer, Early evidence of complexity in lithic economy: Core-axe production, hafting and use at Late Middle Pleistocene site 8-B-11, Sai Island (Sudan). *J. Archaeol. Sci.* **33**, 360–371 (2006).

9. P. Schmidt, T. J. Koch, E. February, Archaeological adhesives made from Podocarpus document innovative potential in the African Middle Stone Age. *Proc. Natl. Acad. Sci. U.S.A.* **119**, e2209592119 (2022).

10. T. Schenck, P. Groom, The aceramic production of Betula pubescens (downy birch) bark tar using simple raised structures. A viable Neanderthal technique? *Anthropol. Sci.* **10**, 19–29 (2018).

11. P. R. B. Kozowyk, M. Soressi, D. Pomstra, G. H. J. Langejans, Experimental methods for the Palaeolithic dry distillation of birch bark: Implications for the origin and development of Neandertal adhesive technology. *Sci. Rep.* **7**, 8033 (2017).

12. P. Schmidt, Steak tournedos or beef Wellington: An attempt to understand the meaning of Stone Age transformative techniques. *Humanit. Soc. Sci. Commun.* **8**, 280 (2021).

13. L. Wadley, M. Mohapi, A segment is not a monolith: Evidence from the Howiesons Poort of Sibudu, South Africa. *J. Archaeol. Sci.* **35**, 2594–2605 (2008).

14. M. Lombard, The gripping nature of ochre: The association of ochre with Howiesons Poort adhesives and Later Stone Age mastics from South Africa. *J. Hum. Evol.* **53**, 406–419 (2007).

15. A. Charrié-Duhaut, G. Porraz, C. R. Cartwright, M. Igreja, J. Connan, C. Poggenpoel, P.J. Texier, First molecular identification of a hafting adhesive in the Late Howiesons Poort at Diepkloof Rock Shelter, Western Cape, South Africa. *J. Archaeol. Sci.* **40**, 3506–3518 (2013).

16. A. M. Zipkin, M. Wagner, K. McGrath, A. S. Brooks, P. W. Lucas, An experimental study of hafting adhesives and the implications for compound tool technology. *PLOS ONE* **9**, e112560 (2014).

17. L. Wadley, Putting ochre to the test: Replication studies of adhesives that may have been used for hafting tools in the Middle Stone Age. *J. Hum. Evol.* **49**, 587–601 (2005).

18. L. Wadley, Compound-adhesive manufacture as a behavioral proxy for complex cognition in the Middle Stone Age. *Curr. Anthropol.* **51**, S111–S119 (2010).

19. T. C. Hauck, J. Connan, A. Charrié-Duhaut, J.-M. Le Tensorer, H. a. Sakhel, Molecular evidence of bitumen in the Mousterian lithic assemblage of Hummal (Central Syria). *J. Archaeol. Sci.* **40**, 3252–3262 (2013).

20. I. Degano, S. Soriano, P. Villa, L. Pollarolo, J. J. Lucejko, Z. Jacobs, K. Douka, S. Vitagliano, C. Tozzi, Hafting of Middle Paleolithic tools in Latium (central Italy): New data from Fossellone and Sant'Agostino caves. *PLOS ONE* **14**, e0213473 (2019).

21. J. Grünberg, H. Gratsch, U. Baumer, J. Koller, Utersuchung der mittelpaläolithischen "Harzreste" von Königsaue, Ldkr. Aschersleben-Stassfurt. *Jahresschrift für mitteldeutsche Vorgeschichte* **81**, 7–38 (1999).

22. P. P. A. Mazza, F. Martini, B. Sala, M. Magi, M. P. Colombini, G. Giachi, F. Landucci, C. Lemorini, F. Modugno, E. Ribechini, A new Palaeolithic discovery: Tar-hafted stone tools in a European Mid-Pleistocene bone-bearing bed. *J. Archaeol. Sci.* **33**, 1310–1318 (2006).

23. R. Dinnis, A. Pawlik, C. Gaillard, Bladelet cores as weapon tips? Hafting residue identification and micro-wear analysis of three carinated burins from the late Aurignacian of Les Vachons, France, *J. Archaeol. Sci.* **36**, 1922–1934 (2009).

24. M. Bradtmöller, A. Sarmiento, U. Perales, M. C. Zuluaga, Investigation of Upper Palaeolithic adhesive residues from Cueva Morín, Northern Spain. *J. Archaeol. Sci. Rep.* **7**, 1–13 (2016).

25. F. Javier Muñoz, V. Rubio, C. Gutiérrez, A. Hernanz, M. Menéndez, THE POINTS FROM EL BUXU CAVE (ASTURIAS, SPAIN): First evidence of adhesive as hafting material in the Solutrean. *J. Archaeol. Sci. Rep.* **49**, 103901 (2023).

26. M. Cârciumaru, R.-M. Ion, E.-C. Niţu, R. Ştefãnescu, New evidence of adhesive as hafting material on Middle and Upper Palaeolithic artefacts from Gura Cheii-Râşnov Cave (Romania). *J. Archaeol. Sci.* **39**, 1942–1950 (2012).

27. P. R. B. Kozowyk, G. H. J. Langejans, G. L. Dusseldorp, M. J. L. T. Niekus, Reply to Schmidt et al.: Interpretation of Paleolithic adhesive production: Combining experimental and paleoenvironmental information. *Proc. Nat. Acad. Sci. U.S.A.* **117**, 4458 (2020).

28. P. Schmidt, M. Rageot, M. Blessing, C. Tennie, The Zandmotor data do not resolve the question whether Middle Paleolithic birch tar making was complex or not. *Proc. Nat. Acad. Sci. U.S.A.* **117**, 4456 (2020), 4457.

29. P. Schmidt, T. J. Koch, C. Tennie, Reply to Paul R.B. Kozowyk: Interpreting the complexity of archaeological adhesives may lead to misconceptions about early humans. *Proc. Nat. Acad. Sci. U.S.A.* **120**, e2300325120 (2023).

30. P. R. B. Kozowyk, Archaeological Podocarpus tar supports the cognitive complexity of Neanderthals. *Proc. Nat. Acad. Sci. U.S.A.* **120**, e2221676120 (2023).

31. M. Tomasello, *The Cultural Origins of Human Cognition* (Harvard Univ. Press, 2009).

32. P. Schmidt, T.J. Koch, M.A. Blessing, F.A. Karakostis, K. Harvati, V. Dresely, A. Charrié-Duhaut, Production method of the Königsaue birch tar documents cumulative culture in Neanderthals. *Archaeol. Anthropol. Sci.* **15**, 84 (2023).

33. L. Slimak, C. Zanolli, T. Higham, M. Frouin, J.-L. Schwenninger, L. J. Arnold, M. Demuro, K. Douka, N. Mercier, G. Guérin, H. Valladas, P. Yvorra, Y. Giraud, A. Seguin-Orlando, L. Orlando, J. E. Lewis, X. Muth, H. Camus, S. Vandevelde, M. Buckley, C. Mallol, C. Stringer, L. Metz, Modern human incursion into Neanderthal territories 54,000 years ago at Mandrin, France. *Sci. Adv.* **8**, eabj9496 (2022).

34. L. Wadley, G. Trower, L. Backwell, F. d'Errico, Traditional glue, adhesive and poison used for composite weapons by Ju/'hoan San in Nyae Nyae, Namibia. Implications for the evolution of hunting equipment in prehistory. *PLOS ONE* **10**, e0140269 (2015).

35. F. Bordes, *Typologie du Paléolithique Ancien et Moyen* (Delmas, 1961).

36. S. Beyries, in *La Main et l'outil. Manches et Emmanchements Préhistoriques,Travaux de La Maison de l'Orient*, D. Stordeur, Ed. (MOM Éditions, 1987), pp. 55–62.

37. V. Rots, Towards an understanding of hafting: The macro- and microscopic evidence. *Antiquity* **77**, 805–815 (2003).

38. V. Rots, *Prehension and Hafting Traces on Flint Tools A Methodology* (Leuven Univ. Press, 2010).

39. I. Levi Sala, Use wear and post-depositional surface modification: A word of caution. *J. Archaeol. Sci.* **13**, 229–244 (1986).

40. M. Blanchard, E. Balan, P. Giura, K. Béneut, H. Yi, G. Morin, C. Pinilla, M. Lazzeri, A. Floris, Infrared spectroscopic properties of goethite: Anharmonic broadening, long-range electrostatic effects and Al substitution. *Phys. Chem. Miner.* **41**, 289–302 (2014).

41. G. N. Kustova, E. B. Burgina, V. A. Sadykov, S. G. Poryvaev, Vibrational spectroscopic investigation of the goethite thermal decomposition products. *Phys. Chem. Miner.* **18**, 379–382 (1992).

42. S. Weigel, D. Stephan, The prediction of bitumen properties based on FTIR and multivariate analysis methods. *Fuel* **208**, 655–661 (2017).

43. B. Hofko, L. Porot, A. Falchetto Cannone, L. Poulikakos, L. Huber, X. Lu, K. Mollenhauer, H. Grothe, FTIR spectral analysis of bituminous binders: Reproducibility and impact of ageing temperature. *Mater. Struct.* **51**, 45 (2018).

44. G. Vázquez-Bautista, F. Chalé-Lara, M. Zapata Torres, M. Meléndez Lira, E. Hernández Rodríguez, E. Valaguez Velázquez, Is sulfur the responsible for color of yellow Chiapas Amber? *Superf. y Vacío* **34**, 211201 (2021).

45. H. D. Ruan, R. L. Frost, J. T. Kloprogge, L. Duong, Infrared spectroscopy of goethite dehydroxylation: III. FT-IR microscopy of in situ study of the thermal transformation of goethite to hematite. *Spectrochim. Acta A Mol. Biomol. Spectrosc.* **58**, 967–981 (2002).

46. A. Olori, P. Di Pietro, A. Campopiano, Preparation of ultrapure KBr pellet: New method for FTIR quantitative analysis. *Int. J. Sci. Res.* **2**, 1015–1020 (2021).

47. V. Beltran, N. Salvadó, S. Butí, T. Pradell, Ageing of resin from Pinus species assessed by infrared spectroscopy. *Anal. Bioanal. Chem.* **408**, 4073–4082 (2016).

48. J.-b. Chen, Q. Zhou, S.-q. Sun, Direct chemical characterization of natural wood resins by temperature-resolved and space-resolved Fourier transform infrared spectroscopy. *J. Mol. Struct.* **1115**, 55–62 (2016).

49. A. Dwivedi, A. K. Pandey, K. Raj, N. Misra, Comparative study of vibrational spectra of two bioactive natural products Lupeol and Lupenone using MM/QM method *Int. J.* **27**, 486304 (2012).

50. R. Fang, R. Littke, L. Zieger, A. Baniasad, M. Li, J. Schwarzbauer, Changes of composition and content of tricyclic terpane, hopane, sterane, and aromatic biomarkers throughout the oil window: A detailed study on maturity parameters of Lower Toarcian Posidonia Shale of the Hils Syncline, NW Germany. *Org. Geochem.* **138**, 103928 (2019).

51. A. Charrié-Duhaut, S. Lemoine, P. Adam, J. Connan, P. Albrecht, Abiotic oxidation of petroleum bitumens under natural conditions. *Org. Geochem.* **31**, 977–1003 (2000).

52. E. M. Aveling, C. I. Heron, Identification of birch bark tar at the Mesolithic site of Star Carr. *Anc. Biomol.* **2**, 69–80 (1998).

53. P. Dietemann, K. v. Miller, C. Höpker, U. Baumer, On the use and differentiation of resins from Pinaceae species in European artworks based on written sources, reconstructions and analysis. *Stud. Conserv.* **64**, S62–S73 (2019).

54. R. T. Mull, Mass estimates by computed tomography: Physical density from CT numbers. *AJR Am. J. Roentgenol.* **143**, 1101–1104 (1984).

55. T. Razi, M. Niknami, F. Alavi Ghazani, Relationship between Hounsfield unit in CT scan and gray scale in CBCT. *J. Dent. Res. Dent. Clin. Dent. Prospects* **8**, 107–110 (2014).

56. E. Yalcin, A. Munir Ozdemir, B. Vural Kok, M. Yilmaz, B. Yilmaz, Influence of pandemic waste face mask on rheological, physical and chemical properties of bitumen. *Construct. Build Mater.* **337**, 127576 (2022).

57. C. Palache, H. Berman, C. Frondel, in *The System of Mineralogy of James Dwight Dana and Edward Salisbury Dana Yale University 1837–1892, Volume I* (John Wiley and Sons, 1944).

58. L. Tydgadt, V. Rots, Stick to it! Mechanical performance tests to explore the resilience of prehistoric glues in hafting. *Archaeometry* **64**, 1252–1269 (2022).

59. P. R. B. Kozowyk, G. H. J. Langejans, J. A. Poulis, Lap shear and impact testing of ochre and beeswax in experimental Middle Stone Age compound adhesives. *PLOS ONE* **11**, e0150436 (2016).

60. P. R. B. Kozowyk, J. A. Poulis, A new experimental methodology for assessing adhesive properties shows that Neandertals used the most suitable material available. *J. Hum. Evol.* **137**, 102664 (2019).

61. J. S. Tomblin, C. C. Yang, P. Harter, Investigation of thick bondline adhesive joints (Report: DOT/FAA/AR-01/33, Department of Transportation, 106, 2001).

62. P. Schmidt, T. J. Koch, C. Berthold, F. Lauxmann, K. G. Nickel, The evolution of strength, elasticity and rupture behaviour of birch tar made with 'double-pot' techniques during tar cooking. *Archaeometry*, **65**, 409–422 (2023).

63. P. Schmidt, M. A. Blessing, T. J. Koch, K. G. Nickel, On the performance of birch tar made with different techniques. *Herit. Sci.* **9**, 140 (2021).

64. P. R. B. Kozowyk, J. A. Poulis, G. H. J. Langejans, Laboratory strength testing of pine wood and birch bark adhesives: A first study of the material properties of pitch. *J. Archaeol. Sci. Rep.* **13**, 49–59 (2017).

65. F. A. Karakostis, G. Hotz, V. Tourloukis, K. Harvati, Evidence for precision grasping in Neandertal daily activities. *Sci. Adv.* **4**, eaat2369 (2018).

66. W. A. Niewoehner, Behavioral inferences from the Skhul/Qafzeh early modern human hand remains. *Proc. Natl. Acad. Sci. U.S.A.* **98**, 2979–2984 (2001).

67. L. Wadley, Compound-adhesive manufacture as a behavioral proxy for complex cognition in the Middle Stone Age. *Curr. Anthropol.* **51**, 111–119 (2010).

68. C. Perreault, P. J. Brantingham, S. L. Kuhn, S. Wurz, X. Gao, Measuring the complexity of lithic technology. *Curr. Anthropol.* **54**, S397–S406 (2013).

69. M. Lombard, M. N. Haidle, Thinking a bow-and-arrow set: Cognitive implications of Middle Stone Age bow and stone-tipped arrow technology. *Camb. Archaeol. J.* **22**, 237–264 (2012).

70. S. Fajardo, J. Kleijn, F. W. Takes, G. H. J. Langejans, Modelling and measuring complexity of traditional and ancient technologies using Petri nets. *PLOS ONE* **17**, e0278310 (2022).

71. S. P. Pratt, Geological position of the bitumen used in asphalte pavements. *J. Geol. Soc.* **2**, 80–81 (1846).

72. A. Morala, *Les silicifications des bassins versants de la Dordogne et de la Vézère: Evaluation des ressources lithiques et implications archéologiques* (Paleo, 2017), 110 pp.

73. A. Sempio, A. Gualtieri, Mineralogy of the Grès de Thiviers (northern Aquitaine, France). *Per. Mineral.* **71**, 65–84 (2002).

74. J.-J. Biteau, A. Le Marrec, M. Le Vot, J.-M. Masset, The Aquitaine Basin. *Pet. Geosci.* **12**, 247–273 (2006).

75. H. Valladas, J. M. Geneste, J. L. Joron, J. P. Chadelle, Thermoluminescence dating of Le Moustier (Dordogne, France). *Nature* **322**, 452–454 (1986).

76. J.-J. Hublin, N. Sirakov, V. Aldeias, S. Bailey, E. Bard, V. Delvigne, E. Endarova, Y. Fagault, H. Fewlass, M. Hajdinjak, B. Kromer, I. Krumov, J. Marreiros, N.L. Martisius, L. Paskulin, V. Sinet-Mathiot, M. Meyer, S. Pääbo, V. Popov, Z. Rezek, S. Sirakova, M.M. Skinner, G.M. Smith, R. Spasov, S. Talamo, T. Tuna, L. Wacker, F. Welker, A. Wilcke, N. Zahariev, S.P. McPherron, T. Tsanova, Initial Upper Palaeolithic *Homo sapiens* from Bacho Kiro Cave, Bulgaria, *Nature* **581**, 299–302 (2020).

77. K. Harvati, C. Röding, A.M. Bosman, F.A. Karakostis, R. Grün, C. Stringer, P. Karkanas, N.C. Thompson, V. Koutoulidis, L.A. Moulopoulos, V.G. Gorgoulis, M. Kouloukoussa, Apidima Cave fossils provide earliest evidence of Homo sapiens in Eurasia. *Nature* **571**, 500–504 (2019).

78. L. Metz, J. E. Lewis, L. Slimak, Bow-and-arrow, technology of the first modern humans in Europe 54,000 years ago at Mandrin, France. *Sci. Adv.* **9**, eadd4675 (2023).

79. A. Picin, A. Moroni, S. Benazzi, in *Updating Neanderthals*, F. Romagnoli, F. Rivals, S. Benazzi, Eds. (Academic Press, 2022), pp. 321–347.

80. P. Villa, W. Roebroeks, Neandertal demise: An archaeological analysis of the modern human superiority complex. *PLOS ONE* **9**, e96424 (2014).

81. J. Speth, News flash: Negative evidence convicts Neanderthals of gross mental incompetence. *World Archaeol.* **36**, 519–526 (2004).

82. O. Bar-Yosef, in *Dynamics of Learning in Neanderthals and Modern Humans Volume 1: Cultural Perspectives*, T. Akazawa, Y. Nishiaki, K. Aokie, Eds. (Springer, 2013), pp. 7–20.

83. A. G. Püntener, S. Moss, Otzi, the iceman and his leather clothes. *Chimia* **64**, 315–320 (2010).

84. E. C. Velliky, P. Schmidt, L. Bellot-Gurlet, S. Wolf, N. J. Conard, Early anthropogenic use of hematite on Aurignacian ivory personal ornaments from Hohle Fels and Vogelherd caves, Germany, *J. Hum. Evol.* **150**, 102900 (2021).

85. I. Watts, in *The Cradle of Language*, R. Botha, C. Knight, Eds. (Oxford Univ. Press, 2009), pp. 62–97.

86. R. F. Rifkin, Assessing the efficacy of red ochre as a prehistoric hide tanning ingredient *Archaeology* **9**, 131–158 (2011).

87. A. Pitarch Martí, J. Zilhão, F. d'Errico, P. Cantalejo-Duarte, S. Domínguez-Bella, J.M. Fullola, G.C. Weniger, J. Ramos-Muñoz, The symbolic role of the underground world among Middle Paleolithic Neanderthals. *Proc. Natl. Acad. Sci. U.S.A.* **118**, e2021495118 (2021).

88. D. Leder, R. Hermann, M. Hüls, G. Russo, P. Hoelzmann, R. Nielbock, U. Böhner, J. Lehmann, M. Meier, A. Schwalb, A. Tröller-Reimer, T. Koddenberg, T. Terberger, A 51,000-year-old engraved bone reveals Neanderthals' capacity for symbolic behaviour. *Nat. Ecol. Evol.* **5**, 1273–1282 (2021).

89. M. C. Langley, C. Clarkson, S. Ulm, Behavioural complexity in Eurasian Neanderthal populations: A chronological examination of the archaeological evidence. *Camb. Archaeol. J.* **18**, 289–307 (2008).

90. E. Lartet, H. Christy, Sur des figures d'animaux gravées ou sculptées et autres produits d'art et d'industrie rapportables aux temps primordiaux de la période humaine. *Rev. Archéol.* **9**, 233–267 (1864).

91. E. Lartet, H. Christy, T. R. Jones, *Reliquae Aquitanicae: Being Contributions to The Archaeology and Palaeontology of Périgord and Adjoining Provinces of Southern France* (Williams & Norgate, 1875).

92. G. de Mortillet, Essai d'une classification des cavernes et des stations sous abri, fondée sur les produits de l'industrie humaine. *Matériaux pour l'histoire primitive et naturelle de l'homme* **4**, 172–179 (1869).

93. G. de Mortillet, Notice sur l'origine du langage. *Congrès international d'anthropologie et d'archéologie préhistoriques* **4**, 286–287 (1869).

94. M. Bourlon, Une fouille au Moustier (Dordogne). *L'homme préhistorique* **7**, 193–204 (1905).

95. M. Bourlon, L'industrie moustérienne au Moustier. *Congrès international d'anthropologie et d'archéologie préhistoriques* **13**, 287–322 (1906).

96. O. Hauser, Fouilles scientifiques à la Micoque, à Laugerie-Basse et au Moustier. *L'homme préhistorique* **6**, 40–48 (1908).

97. O. Hauser, *Le Périgord préhistorique, guide pour les excursions dans les Vallées de la Vézère et de la Dordogne et pour l'étude de leurs stations préhistoriques* (Réjou, 1911).

98. D. Peyrony, Après une grande crue préhistorique de la Vézère. *Revue de géographie commerciale* **4**, 123–127 (1914).

99. D. Peyrony, Le Moustier, ses gisements, ses industries, ses couches géologique. *Revue anthropologie* **40**, 48–76 (1930).

100. A. Hoffmann, Le Moustier und Combe Capelle. *Die altsteinzeitlichen Funde des Schweizer Archäologen Otto Hauser. Museum für Vor- und Frühgeschichte Bestandskatalog, Band 9* (Staatliche Museen zu Berlin – Preußischer Kulturbesitz, 2003).

101. R. Busch, Dr. Alfred Tode Braunschweiger Landesarchäologe i. R. verstorben. *Nachrichten aus Niedersachsens Urgeschichte* **65**, 235–236 (1996).

102. R. Drößler, *Flucht aus dem Paradies. Leben, Ausgrabungen und Entdeckungen Otto Hausers* (Mitteldeutscher Verlag, 1988).

103. R. Drössler, S. Drössler, M. Freyberg, The Swiss archaeologist Otto Hauser. His skeleton findings and assumptions concerning the Evolution of man. *Anthropol. Anz.* **64**, 97–123 (2006).

104. A. Debénath, H. L. Dibble, *Handbook of Paleolithic Typology Lower and Middle Paleolithic of Europe* (University of Pennsylvania Press, 1993).

105. V. Rots, Prehensile wear on flint tools. *Lithic Technol.* **29**, 7–32 (2004).

106. M. E. Mansur, Microwear analysis of natural and use striations: New clues to the mechanisms of striation formation. *Studia Praehistorica Belgica Leuven* **2**, 213–233 (1982).

107. T. A. Del Bene, in *Lithic Use-Wear Analysis*, B. Hayden, Ed. (Academic Press, 1979), pp. 167–177.

108. L. H. Keeley, *Experimental Determination of Stone Tool Uses: A Microwear Analysis* (University of Chicago Press, 1980).

109. G. H. Odell, F. Odell-Vereecken, Verifying the relationships of lithic use wear assessments by 'blind tests': The low-power approach. *J. Field Archaeol.* **7**, 87–120 (1980).
